# Diacylglycerol enantiomer selectivity of diacylglycerol acyltransferases highlights metabolic specialization in triacylglycerol synthesis across the tree of life

**DOI:** 10.1042/BSR20260190

**Published:** 2026-06-22

**Authors:** Prasad Parchuri, Jay Shockey, Timothy P. Durrett, Philip D. Bates

**Affiliations:** 1 Institute of Biological Chemistry, Washington State University, Pullman, WA 99164, U.S.A.; 2 Current address: Division of Biology, Kansas State University, Manhattan, KS 66506, U.S.A.; 3 United States Department of Agriculture, Agricultural Research Service, Southern Regional Research Center, New Orleans, LA 70124, U.S.A.; 4 Department of Biochemistry and Molecular Biophysics, Kansas State University, Manhattan, KS 66506, U.S.A.

**Keywords:** Arabidopsis thaliana, diacylglycerol, diacylglycerol acyltransferase, lipid metabolism, lipids, triglycerides

## Abstract

Triacylglycerols are the major energy storage lipids in plants, animals, and microorganisms, and are predominantly produced by acyl-CoA:diacylglycerol (DAG) acyltransferases (DGATs). Two enantiomers of the DAG substrate, *sn*-1,2 and *sn*-2,3, can be produced by different biological mechanisms; however, little is known about which species produce each enantiomer, the selectivity of DGAT isoforms for either enantiomer, or whether DGAT enantiomer selectivity varies across organisms. Here, DAG enantiomer selectivity of DGAT1 and DGAT2 was measured from eight seed plants, two mammals, one oleaginous yeast, and one photosynthetic microalga using enantiomer-specific *in vitro* DGAT assays. Across most plants, DGAT1 favored *sn*-1,2-DAG, whereas DGAT2 preferentially utilized *sn*-2,3-DAG. However, there were several exceptions. Mammalian DGAT1, DGAT2, and microbial DGAT1s efficiently used both DAG enantiomers, while microbial DGAT2s had unique selectivity. The selectivity of several DGATs for combined acyl-CoA and DAG enantiomer molecular species were also evaluated for biotechnical applications. Therefore, DGAT DAG enantiomer selectivity is common yet strongly dependent on lineage and isoform and likely shaped in part by species-specific metabolic context of triacylglycerol synthesis, turnover, and remodeling. This work expands our understanding of DGAT function and establishes a foundation for leveraging enantiomer-selective acyltransferases in metabolic engineering of tailored lipid products.

## Introduction

Triacylglycerols (TAGs) are the most energy dense form of carbon storage utilized across the tree of life [1]. In mammals, TAGs are stored in adipose tissue, where they serve as the primary energy source during periods of food deprivation or between meals [2]. Many mammals consume TAG-rich plant seeds as a calorie-dense nutrient source and to obtain essential polyunsaturated fatty acids (PUFAs) that cannot be synthesized endogenously [3,4]. Additionally, the large diversity of fatty acid (FA) structures found in plants make many plant TAGs a valuable resource for chemical and biofuel industries [5]. Both the nutritional and industrial importance of TAGs emphasizes the need to understand the mechanisms of TAG biosynthesis in different organisms. Furthermore, understanding the diversity within TAG biosynthetic mechanisms in different species provides a myriad of possible genetic tools to engineer designer TAG compositions within plants or microorganisms for food, fuel, or industrial feedstocks within the bioeconomy.

In mammals, excess carbohydrates and amino acids are converted into FAs through *de novo* lipogenesis and esterified into TAGs in the liver for storage or export [6]. Mammalian cells initiate TAG formation through three distinct routes: acylation of glycerol-3-phosphate in the mitochondria or endoplasmic reticulum (ER), conversion of dihydroxyacetone phosphate to acylated intermediates in peroxisomes, or acylation of *sn*-2-monoacylglycerol (MAG) within the ER [7]. Dietary TAGs predominantly enter metabolism through the third route where TAG is initially hydrolyzed in the small intestine into *sn*-2-MAG and non-esterified fatty acids (NEFA), which are absorbed by enterocytes, re-esterified into TAGs via the MAG pathway, and packaged into chylomicrons for lymphatic transport [8,9]. After transport to peripheral tissues, chylomicron TAGs are hydrolyzed, enabling FA uptake by muscle tissue as an energy source, or by adipose tissue to re-synthesize TAG for storage. The liver also recycles chylomicron remnants to synthesize TAGs and assemble very-low-density lipoproteins for TAG delivery to adipose tissue [10]. In adipose tissue, TAGs are stored in lipid droplets and mobilized during fasting via lipolysis to provide energy and metabolic intermediates [2]. Thus, TAG metabolism in mammals is tightly regulated and highly dynamic, with continual flux between anabolic storage and catabolic mobilization in response to cellular/tissue/organismal nutrient and energy status.

In photosynthetic organisms, most TAG accumulation is less dynamic and predominantly controlled through developmental processes. In unicellular microalgae under optimal growth conditions little TAG accumulates. However, under nutrient stress (e.g. limited nitrogen, phosphorus, etc.) growth is stopped and TAG rapidly accumulates as a carbon and energy store, and as a sink for excess reductant produced prior to breakdown of photosynthetic membranes [11,12]. When environmental conditions are sufficient to re-initiate growth, the stored TAG is broken down to provide carbon, energy, and FAs for rapid reassembly of photosynthetic membranes [13,14]. In seed bearing land plants TAG predominantly accumulates as a stable carbon and energy store during seed and pollen development [5,15,16]. Seeds can remain dormant from weeks to hundreds of years. During eventual seed germination the stored TAG is completely broken down, providing energy and carbon skeletons for plant growth until photosynthetic competency is reached [17]. Similarly, pollen TAG is metabolized providing FAs for rapid pollen tube growth during plant fertilization [15]. Therefore, TAG accumulation and degradation in photosynthetic organisms are controlled developmentally and are generally considered less dynamic than in mammals.

The degree of TAG biosynthetic dynamics is partly explained by substrate availability and substrate utilization of the TAG synthesizing enzymes. TAG is synthesized predominantly by acyl-CoA: diacylglycerol acyltransferases (DGATs). Most organisms have at least one copy of the structurally unrelated DGAT1 and/or DGAT2 isozymes [1,5]. TAG FA composition is highly dependent on the composition of both the acyl-CoA and diacylglycerol (DAG) substrate pools and the molecular species substrate selectivity of each enzyme [18–20]. *In vitro* DGAT assays have been very valuable for determining the substrate selectivity of DGATs, which has been characterized for type-1 (DGAT1) and type-2 (DGAT2) enzymes from many species [1,18,19,21]. Species-specific DGAT substrate selectivities have also been essential components of bioengineering approaches to produce unique TAG FA compositions in plants and microorganisms [22,23]. Despite the large amount of knowledge on DGAT substrate selectivity, extremely little is known about specificity for either the *sn*-1,2- or *sn*-2,3- enantiomers of DAG.

Glycerolipids are synthesized stereospecifically from *sn*-glycerol-3-phosphate and the acyl-CoA dependent acyltransferases of the Kennedy pathway producing phosphatidic acid (PA, 1,2-diacyl-*sn*-glycerol-3-phosphate). PA dephosphorylation produces *sn*-1,2-DAG that can be utilized for synthesis of membrane lipids or TAG. However, lipases involved in TAG turnover can hydrolyze FAs from either the *sn*-1 or *sn*-3 positions of TAG. Thus, the intermediates of TAG turnover create substrate pools that can contain a mixture of *sn*-1,2-DAG and *sn*-2,3-DAG enantiomers [20,24,25]. Likewise, mammalian MAG acyltransferases involved in the resynthesis of ingested TAG in the liver can produce both *sn*-1,2- and *sn*-2,3-DAG [24,26]. Therefore, the dynamic TAG metabolism in mammals likely requires that DGAT1 and/or DGAT2 can utilize both DAG enantiomers. *In vitro* assays of human DGATs with racemic mixtures of both *sn*-1,2- and *sn*-2,3-DAG substrates support this assumption [20].

Plant seed TAG synthesis has been typically characterized as either utilizing *de novo sn*-1,2-DAG from the Kennedy pathway in plants such as castor bean (*Ricinus communis*) or *Cuphea* species, or from *sn*-1,2-DAG produced by removal of the phosphocholine headgroup from the membrane lipid phosphatidylcholine (PC; 1,2-diacyl-sn-glycero-3-phosphocholine) in species such as *Arabidopsis thaliana*, soybean (*Glycine max*), and *Camelina sativa* [5,27–31]. Since PC is the site of FA desaturation in the plant ER, PC-derived DAG contributes to the enrichment of PUFAs in TAG [5]. Both *de novo* DAG and PC-derived DAG are the *sn*-1,2 stereoisomer, and given the relatively static nature of TAG biosynthesis during seed development, it has been widely assumed that plant DGATs predominantly utilize *sn*-1,2-DAG as the acyl acceptor. However, the recent discovery of TAG biosynthesis via TAG remodeling in the plant *Physaria fendleri* challenged this assumption and indicated that plant DGATs can be selective for either *sn*-1,2- or *sn*-2,3-DAG enantiomers [19,32].

TAG remodeling in *P. fendleri* (*Pfe*) starts with initial TAG synthesis by *Pfe*DGAT1 utilizing *sn*-1,2-PC-derived DAG and producing a TAG molecular species with an unusual hydroxylated FA (lesquerolic acid) at the *sn*-3 position. Subsequent lipase activity at the TAG *sn*-1 position removes a common FA and generates the *sn*-2,3-enantiomer of DAG. *Pfe*DGAT2, that was demonstrated to have a strong preference for *sn*-2,3-DAG, transfers a second lesquerolic acid to the *sn*-1 position producing a final TAG molecular species that contains two unusual hydroxy fatty acids (HFAs). Thus, through TAG remodeling *P. fendleri* changes the oil FA composition after initial synthesis, a process that likely evolved as a way to exclude unusual FAs from membrane lipid intermediate pools to reduce the risk of negative effects on membrane structure/function [19,32]. Interestingly, these results imply that plant DGAT1 and DGAT2 can be selective for different DAG enantiomers. Thus, the discovery of TAG remodeling during the oil accumulation phase of seed development implies that plant TAG accumulation can also be dynamic, involving cycles of TAG synthesis, partial degradation, and resynthesis that rely on enantiomer-selective enzymes to control final TAG FA composition.

Currently, it is unknown if TAG remodeling occurs in other plants or other TAG-accumulating organisms. Likewise, it is unclear if DGATs across the tree of life have selectivity for specific DAG enantiomers, and if so, how these properties impact lipid metabolism. The demonstration of selectivity of other plant DGATs for *sn*-2,3-DAG may indicate more complex TAG dynamics during seed oil accumulation than previously known, and the presence of TAG remodeling pathways in plants other than *P. fendleri.* Additionally, understanding which species have enantiomer-selective DGATs can broaden our understanding of the unique roles of DGAT1 and DGAT2 from different species, and strengthen the ‘genetic toolbox’ required for production of designer TAG molecular species. Therefore, in this study we determined the enantiomer specificity of DGAT1 and DGAT2 from eight land plant species, two mammals, and two microorganisms (one photosynthetic and one heterotrophic), to assess whether DAG enantiomer specificity influences TAG biosynthesis across phylogenetically and metabolically diverse systems. Our results demonstrate distinct differences in DAG enantiomer selectivity between plant and non-plant DGAT enzymes. Importantly, we also evaluated DAG enantiomer selectivity of *Arabidopsis* DGAT1 and DGAT2 with unusual FAs, which highlighted additional biochemical constraints for accumulation of HFA in engineered lines. This work expands our understanding of DGAT function and provides a framework for exploiting enantiomer-selective acyltransferases in synthetic biology and metabolic engineering of tailored lipid products.

## Results

### Phylogenetic and metabolic diversity of selected plant and non-plant species reflects the evolutionary landscape of TAG metabolism

**Figure 1:**
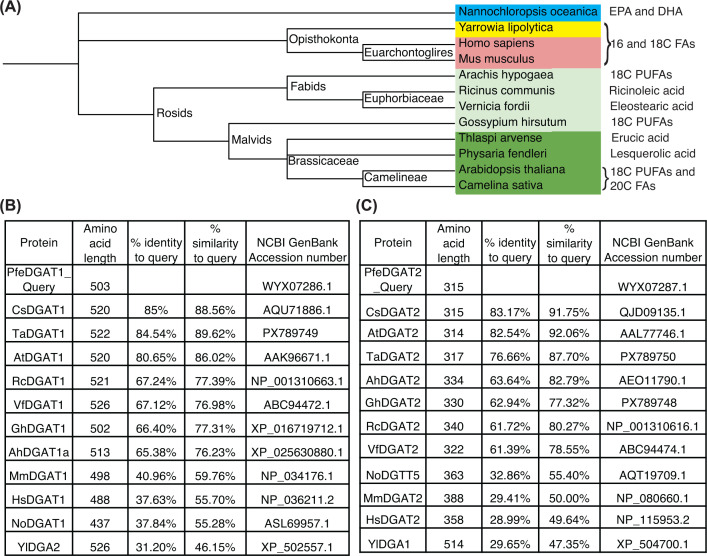
Phylogenetic relationships of selected species and sequence comparison of DGAT1 and DGAT2 proteins analyzed in this study (**A**) Phylogenetic tree illustrating the evolutionary placement of species representing major taxonomic groups. Species belonging to Brassicaceae, non-Brassicaceae dicots, mammals, yeast, and algae are highlighted in dark green, light green, pink, yellow, and blue, respectively. The predominant unusual or characteristic FA produced by each species is indicated adjacent to its name. Abbreviations: fatty acids (FAs); polyunsaturated fatty acid (PUFAs); eicosapentaenoic acid (EPA); and docosahexaenoic acid (DHA). Amino acid sequence length, identity, and similarity of DGAT1 (**B**) and DGAT2 (**C**) homologs relative to *P. fendleri* DGAT1 (PfeDGAT1), which was used as the query sequence for BLAST and multiple sequence alignment (MSA).

To investigate the evolutionary and functional breadth of DAG enantiomer selectivity in TAG biosynthesis, we selected twelve representative species spanning a broad phylogenetic range (Figure 1) and diverse lipid metabolic contexts. The dataset includes eight land plant species, two mammals, one heterotrophic fungus, and one photosynthetic microalga. These organisms represent key taxonomic and metabolic lineages including: oilseed-producing Brassicaceae species *A. thaliana, C. sativa*, and *Thlaspi arvense* (pennycress); other eudicot oil seed plants with diverse oil compositions *Gossypium hirsutum* (cotton), *Vernicia fordii* (tung), *R. communis* (castor), and *Arachis hypogaea* (peanut); the mammals *Homo sapiens* and *Mus musculus*; the oleaginous yeast *Yarrowia lipolytica*; and the marine microalga *Nannochloropsis oceanica*.

These species were chosen to encompass a wide range of TAG biosynthetic strategies and FA compositions, including producers of 18-carbon, 20-carbon, and 22-carbon PUFAs, very-long-chain monounsaturated FAs, HFAs, cyclopropane FAs, and other unusual lipid structures (see Figure 1). Since DAG enantiomer selectivity was first described in *P. fendleri*, we performed MSA of *Pfe*DGAT1 and *Pfe*DGAT2 against homologs from all species examined in this study. Across the full dataset, DGAT1 homologs shared 46%–89% amino acid similarity with *Pfe*DGAT1, while DGAT2 homologs shared 47%–91% similarity with *Pfe*DGAT2 (Figure 1B,C). In both cases, the highest similarity values were observed in Brassicaceae species followed by non-Brassicaceae species, whereas the lowest similarities occurred in mammalian and microbial lineages. Comparative sequence analysis revealed that the fatty acyl–CoA binding motif of DGAT1 is strongly conserved across plants, mammals, algae, and microbial species (Supplementary Figure S1) [22,33,34]. In contrast, conservation of the DAG-binding motif is clade-dependent, suggesting lineage-specific adaptations in DAG recognition. DGAT2 proteins across species share several highly conserved motifs, including the PH, PR, GGE, VPFG, and G motifs (supplementary Figure S2) [22,35]. Overall, this degree of phylogenetic and metabolic diversity provides a robust framework for comparing the enantiomeric substrate preferences of DGAT1 and DGAT2 enzymes across species that vary not only in their evolutionary lineage but also in the complexity and specialization of their TAG metabolic pathways. By evaluating DGAT function across this taxonomically and functionally diverse panel, we aimed to uncover patterns of conservation and divergence in DAG enantiomer selectivity, and to determine whether these preferences correlate with evolutionary relationships, lipid metabolic adaptations (such as TAG remodeling), or enzyme isoform identity.

### DGAT1 and DGAT2 from Brassicaceae species exhibit distinct DAG enantiomer selectivity

**Figure 2:**
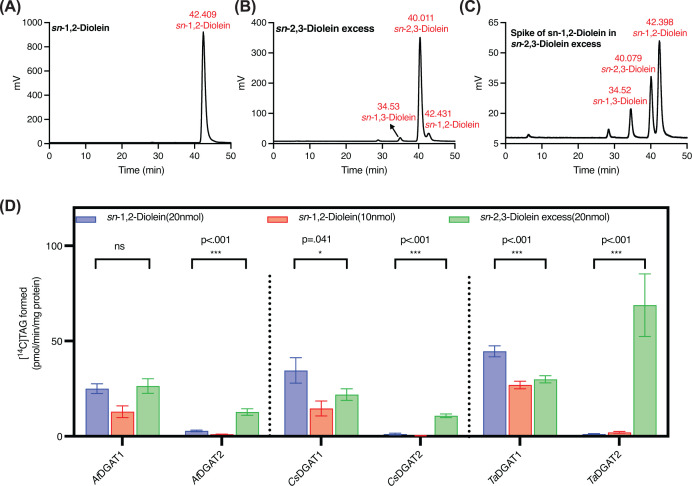
*DGAT1 and DGAT2* enzymes from Brassicaceae species, *Arabidopsis* (*At*), *Camelina* (*Ca*), and *Thlaspi* (*Ta*) show differential specificities toward DAG enantiomers in TAG biosynthesis (**A–C**) High-performance liquid chromatography (HPLC) chromatograms showing the separation of diolein enantiomers. (**A**) Commercial *sn*-1,2-diolein shows a single peak, confirming 100% *sn*-1,2 identity. (**B**) Partial hydrolysis of triolein with *Rhizomucor miehei* TAG lipase produces a mixture enriched in *sn*-2,3-diolein (∼89%), with minor *sn*-1,2-DAG (∼8%) and *sn*-1,3-DAG (∼2.8%), which is designated as *sn*-2,3-diolein excess. (**C**) Spike-in of *sn*-1,2-diolein into the *sn*-2,3-diolein excess mixture confirms peak identities. (**D**) Enzyme assays were performed using [^14^C]18:1-CoA (1 nmol) as the acyl donor and either *sn*-1,2-diolein at 20 nmol (blue) or 10 nmol (red), or 20 nmol *sn*-2,3-diolein excess (green) as acyl acceptors. Bars represent the mean ± standard deviation (SD) of three technical replicates. Statistical significance was determined by one-way ANOVA followed by post-hoc tukey’s using GraphPad Prism. ns, not significant.

To examine the enantiomeric substrate preferences of DGAT enzymes involved in TAG biosynthesis, we conducted enantiomer-specific DGAT assays using DGAT1 and DGAT2 isoforms from three Brassicaceae species that differ in their seed FA profiles, *A. thaliana* (*At*), *C. sativa* (*Cs*), and *T. arvense* (*Ta*) (Figure 2). These species represent a model plant and two closely related oilseed crops that predominantly accumulate 18-carbon PUFAs and very-long-chain FAs (≥C20), providing a metabolically diverse framework to evaluate differences in DAG enantiomer selectivity among related DGAT isoforms.

To determine DAG enantiomer selectivity, each DGAT gene was expressed in the TAG-deficient yeast quadruple mutant H1246 [36] that is unable to produce TAG. However, only the *sn*-1,2-DAG enantiomer is commercially available, therefore the *sn*-1 FA selective *Rhizomucor miehei* TAG lipase (25) was used to partially cleave [^12^C]triolein to a racemic mixture enriched in *sn*-2,3-DAG. The enantiomeric composition of purchased and synthesized DAG substrates used in the assays was verified by chiral HPLC analysis. Commercial *sn*-1,2-diolein yielded a single sharp peak, confirming 100% purity of the *sn*-1,2 isomer (Figure 2A). In contrast, the racemic DAG mixture generated via partial TAG hydrolysis contained approximately 89.5% *sn*-2,3-DAG, 7.7% *sn*-1,2-DAG, and 2.3% *sn*-1,3-DAG, based on peak integration (Figure 2B). This mixture was thus designated ‘*sn*-2,3-diolein excess’. The *sn*-1,2- and *sn*-2,3-DAG retention time are closely spaced, therefore, to confirm enantiomer separation and thus identify the *sn*-2,3-DAG, the ‘*sn*-2,3-diolein excess’ sample was spiked with the known *sn*-1,2-diolein standard. As predicted the enantiomers separated confirming the peak identities (Figure 2C).

Microsomal DGAT assays were conducted using [^14^C]18:1-CoA as the acyl donor and either *sn*-1,2-diolein (10 or 20 nmol) or *sn*-2,3-diolein excess (20 nmol) as the acyl acceptor. Reactions with two concentrations of *sn*-1,2-DAG were performed to compare TAG production at different substrate levels and establish a quantitative baseline for comparison. In all DGAT1 and DGAT2 assays, TAG production with 20 nmol *sn*-1,2-DAG was nearly double of that with 10 nmol *sn*-1,2-DAG, indicating the DAG substrate was not limiting under the assay conditions (Figure 2D). This relationship provided a reference for interpreting reactions using the *sn*-2,3-DAG excess substrate. Given this substrate contained only ∼7.7% *sn*-1,2-DAG (Figure 2B), TAG production limited strictly to *sn*-1,2-DAG utilization would be expected to reach no more than ∼7.7% of that formed in the 20 nmol *sn*-1,2-DAG reaction. Therefore, any TAG accumulation from *sn*-2,3-DAG excess substrate substantially exceeding this expected value indicates that the enzyme can also acylate *sn*-2,3-DAG. The extent to which TAG production surpasses the predicted *sn*-1,2-DAG contribution provides a direct quantitative measure of the enzyme’s relative activity and selectivity toward the *sn*-2,3 DAG enantiomer.

Among Brassicaceae DGAT1 isoforms, *At*DGAT1 produced 19.4 and 36.9 pmol min^−1^ mg^−1^ TAG with 10 and 20 nmol *sn*-1,2-DAG, respectively, and 44.6 pmol min^−1^ mg^−1^ with *sn*-2,3-DAG excess substrate. Given that the *sn*-2,3-DAG substrate contained only ∼7.7% *sn*-1,2-DAG, approximately 3.4 pmol min^−1^ mg^−1^ of TAG in the *sn*-2,3-DAG excess reaction could arise from *sn*-1,2-DAG. The remaining >40 pmol min^−1^ mg^−1^ TAG therefore originate from *sn*-2,3-DAG utilization, confirming that *At*DGAT1 can acylate both DAG enantiomers (Figure 2D), which is distinct from prior *P. fendleri* DGAT1 results [19]. In contrast, *Cs*DGAT1 and *Ta*DGAT1 displayed a statistically significant stronger preference for *sn*-1,2-DAG (Figure 2D). *Cs*DGAT1 produced 34.5 pmol min^−1^ mg^−1^ TAG with 20 nmol *sn*-1,2-DAG and 21.8 pmol min^−1^ mg^−1^ with *sn*-2,3-DAG excess substrate, while *Ta*DGAT1 yielded 44.6 and 29.9 pmol min^−1^ mg^−1^, respectively. Based on the enantiomeric composition of the *sn*-2,3-DAG excess substrate, only ∼1.7–2.7 pmol min^−1^ mg^−1^ of TAG could derive from the *sn*-1,2 fraction, whereas ∼19–27 pmol min^−1^ mg^−1^ originate from *sn*-2,3-DAG. This corresponds to an approximately 1.5–1.6-fold higher activity with *sn*-1,2-DAG compared to *sn*-2,3-DAG, indicating both *Cs*DGAT1 and *Ta*DGAT1 have a higher selectivity for *sn*-1,2-DAG than *sn*-2,3-DAG.

Across all three Brassicaceae species, DGAT2 isoforms displayed the opposite trend, consistently exhibiting strong preferences for *sn*-2,3-DAG over *sn*-1,2-DAG, although the magnitude of selectivity varied (Figure 2D). *At*DGAT2 produced 12.7 pmol min^−1^ mg^−1^ TAG with the *sn*-2,3-DAG excess substrate but only 2.8 pmol min^−1^ mg^−1^ with 20 nmol *sn*-1,2-DAG representing an ∼4.5-fold preference for *sn*-2,3-DAG. This trend was even more pronounced in *C. sativa* and *T. arvense*. *Cs*DGAT2 formed 10.8 pmol min^−1^ mg^−1^ TAG from *sn*-2,3-DAG excess substrate compared to 1.2 pmol min^−1^ mg^−1^ from *sn*-1,2-DAG (∼9-fold difference), while *Ta*DGAT2 showed the highest selectivity, producing 68.8 pmol min^−1^ mg^−1^ with *sn*-2,3-DAG excess substrate but only 1.2 pmol min^−1^ mg^−1^ with *sn*-1,2-DAG, an ∼57-fold difference. Because the *sn*-2,3-DAG excess substrate contains only ∼7.7% *sn*-1,2-DAG and ∼89.5% *sn*-2,3-DAG, these elevated TAG formation rates far exceed the expected contribution from *sn*-1,2-DAG alone, confirming that DGAT2s from all three species selectively acylate the *sn*-2,3-DAG enantiomer. The DGAT2 selectivity for *sn*-2,3-DAG here are similar to what was originally found in the related Brassicaceae species *P. fendleri* [19], but the strength of the selectivity is much higher for these Brassicaceae enzymes. Collectively, these data demonstrate that Brassicaceae DGAT2s are functionally tuned toward *sn*-2,3-DAG utilization, suggesting an evolutionarily conserved specialization that may support TAG remodeling processes during seed oil biosynthesis in species other than *P. fendleri*.

Taken together, these findings reveal distinct stereochemical preferences between DGAT isoforms in Brassicaceae. DGAT1 enzymes from *P. fendleri, C. sativa*, and *T. arvense* exhibit a strong bias toward *sn*-1,2-DAG, with *At*DGAT1 uniquely capable of utilizing both enantiomers, whereas all Brassicaceae DGAT2 enzymes tested indicate high selectivity for *sn*-2,3-DAG. This functional divergence suggests complementary roles of DGAT1 and DGAT2 in the stereochemical regulation of TAG biosynthesis and remodeling during seed oil accumulation.

### Differential recognition of DAG enantiomers by DGAT isoforms from non-Brassicaceae oil crops

**Figure 3:**
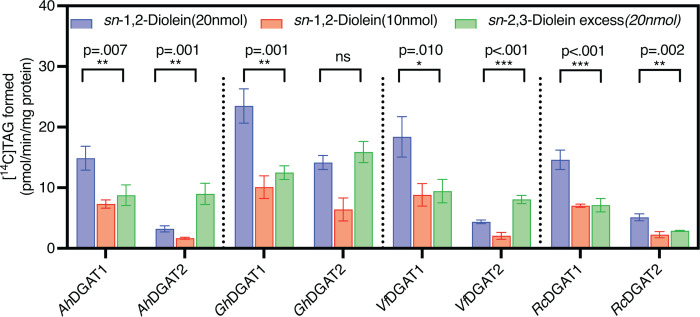
*DGAT1 and DGAT2* enzymes from non-Brassicaceae *A. hypogaea, G. hirsutum, V. fordii, R. communis* show differential specificities toward DAG enantiomers in TAG biosynthesis Enzyme assays were performed using [^14^C]18:1-CoA (1 nmol) as the acyl donor and either *sn*-1,2-diolein at 20 nmol (blue) or 10 nmol (red), or 20 nmol *sn*-2,3-diolein excess (green; ∼89% *sn*-2,3-diolein, ∼8% *sn*-1,2-diolein and ∼2.8% of *sn*-1,3-diolein) as acyl acceptors. Bars represent the mean ± SD of three technical replicates. Statistical significance was determined by one-way ANOVA followed by post-hoc tukey’s using GraphPad Prism. ns, not significant.

To extend our analysis beyond Brassicaceae plants, we examined DGAT1 and DGAT2 isoforms from four non-Brassicaceae oilseed crop species (Figure 3): *A. hypogaea* (*Ah*), *G. hirsutum* (*Gh*), *V. fordii* (*Vf*), and *R. communis* (*Rc*), each characterized by distinct seed oil compositions [37]. *A. hypogaea* and *G. hirsutum* primarily accumulate 18-carbon PUFAs, with *G. hirsutum* also producing small amounts of cyclopropane FAs. In contrast, *V. fordii* and *R. communis* predominantly (>80%) accumulate the unusual FAs α-eleostearic acid and ricinoleic acid, respectively, making them useful models for investigating the relationship between FA specialization and DGAT substrate selectivity. DGAT enantiomer assays were carried out as described above.

For DGAT1 isoforms from non-Brassicaceae species, TAG accumulation with 20 nmol *sn*-1,2-DAG was consistently higher than with the *sn*-2,3-DAG excess substrate, indicating a strong preference for the *sn*-1,2 DAG configuration (Figure 3). *Ah*DGAT1 produced 14.86 pmol min^−1^ mg^−1^ TAG with 20 nmol *sn*-1,2-DAG and 8.75 pmol min^−1^ mg^−1^ with *sn*-2,3-DAG excess, a 1.7-fold preference for *sn*-1,2-DAG. Similarly, *Gh*DGAT1 showed 23.48 versus 12.49 pmol min^−1^ mg^−1^ (1.9-fold), *V. fordii* (*Vf*DGAT1) 18.39 versus 9.43 pmol min^−1^ mg^−1^ (2.0-fold), and *Rc*DGAT1 14.60 versus 7.12 pmol min^−1^ mg^−1^ (2.1-fold). Because only ∼7.7% of the *sn*-2,3-DAG substrate corresponds to the *sn*-1,2 isomer, the observed TAG levels from the racemic reactions exceed this fraction, suggesting that DGAT1s retain modest capacity to utilize *sn*-2,3-DAG. Nonetheless, all four DGAT1s showed significantly greater activity with *sn*-1,2-DAG, confirming a conserved enantiomeric bias toward the *sn*-1,2 DAG configuration.

In contrast, DGAT2 isoforms from non-Brassicaceae species displayed diverse and species-specific patterns of enantiomer selectivity (Figure 3). *Ah*DGAT2 produced 3.20 pmol min^−1^ mg^−1^ TAG with *sn*-1,2-DAG and 8.98 pmol min^−1^ mg^−1^ with *sn*-2,3-DAG excess, a 2.8-fold increase, indicating clear preference for *sn*-2,3-DAG. *Vf*DGAT2 showed 4.37 versus 8.06 pmol min^−1^ mg^−1^ (1.8-fold) under the same conditions, suggesting appreciable *sn*-2,3-DAG utilization. By contrast, *Gh*DGAT2 produced nearly equivalent levels 14.15 pmol min^−1^ mg^−1^ with *sn*-1,2-DAG and 15.89 pmol min^−1^ mg^−1^ with *sn*-2,3-DAG excess indicating little or no enantiomer selectivity. Conversely, *Rc*DGAT2 yielded 5.11 pmol min^−1^ mg^−1^ with *sn*-1,2-DAG but only 2.88 pmol min^−1^ mg^−1^ with *sn*-2,3-DAG excess (1.8-fold preference for *sn*-1,2-DAG), displaying the opposite trend.

Together, these results indicate that while non-Brassicaceae DGAT1 enzymes universally favor *sn*-1,2-DAG, DGAT2 isoforms exhibit broader diversity in stereochemical preference. *Ah*DGAT2 and *Vf*DGAT2 have strong activity toward *sn*-2,3-DAG, *Gh*DGAT2 is non-selective, and *Rc*DGAT2 preferentially utilizes *sn*-1,2-DAG. This diversity underscores that DGAT2s possess greater flexibility in accommodating DAG enantiomers, reflecting species-specific adaptations in TAG biosynthetic mechanisms.

### Mammalian DGAT1 and DGAT2 efficiently utilize both DAG enantiomers

To determine whether plant DGAT DAG enantiomer selectivity extends to animals, we evaluated the activity of DGAT1 and DGAT2 isoforms from two mammalian species *H. sapiens* (*Hs*) and *M. musculus* (*Mm*). These species were selected to represent a physiologically and evolutionarily relevant comparison: *H. sapiens* for the importance of lipid metabolism in various human diseases and disorders, and *M. musculus* as a widely used experimental mammalian model with conserved lipid biosynthetic pathways. The DGAT DAG enantiomer assays were carried out with each DGAT gene expressed in yeast microsomes as described above.

**Figure 4:**
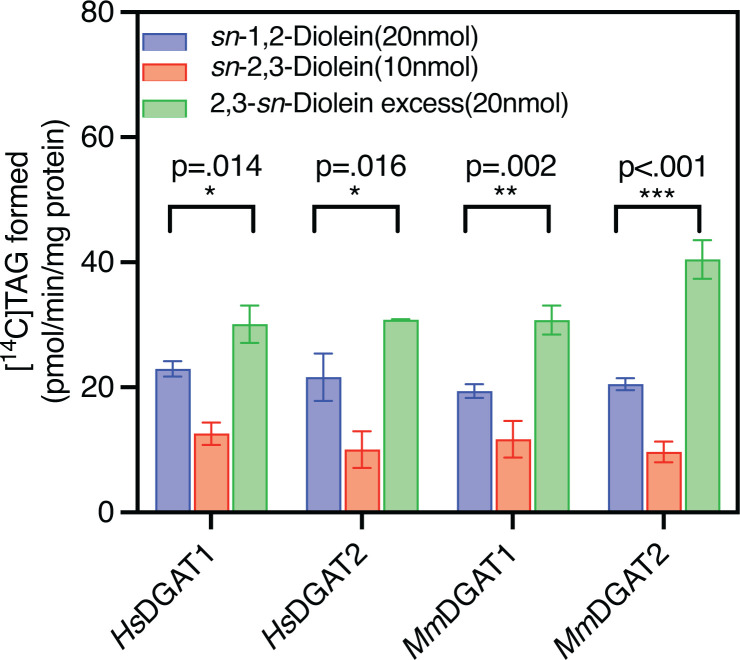
*DGAT1 and DGAT2* enzymes from mammals (humans and mouse) utilize both DAG enantiomers Enzyme assays were performed using [^14^C]18:1-CoA (1 nmol) as the acyl donor and either *sn*-1,2-diolein at 20 nmol (blue) or 10 nmol (red), or 20 nmol sn-2,3-diolein excess (green; ∼89% *sn*-2,3-diolein, ∼8% *sn*-1,2-diolein and ∼2.8% of *sn*-1,3-diolein) as acyl acceptors. Bars represent the mean ± SD of three technical replicates. Statistical significance was determined by one-way ANOVA followed by post-hoc tukey’s using GraphPad Prism. ns, not significant.

All mammalian DGATs had strong activity with both DAG enantiomers (Figure 4). For both human and mouse enzymes, TAG accumulation from 20 nm *sn*-2,3-DAG excess significantly exceeded that from 20 nmol *sn*-1,2-DAG, suggesting that mammalian DGAT1 and DGAT2 enzymes both efficiently utilize *sn*-2,3-DAG (Figure 4). In humans, *Hs*DGAT1 produced 22.94 pmol/min/mg and HsDGAT2 21.63 pmol/min/mg with *sn*-1,2-DAG, but both exhibited ∼30–31 pmol/min/mg activity with *sn*-2,3-DAG excess, representing an ∼1.4-fold increase, well beyond what would be expected from the *sn*-1,2-DAG content alone. Similar trends were observed with the mouse enzymes: *Mm*DGAT1 and *Mm*DGAT2 produced 19.41 and 20.51 pmol/min/mg TAG with *sn*-1,2-DAG, respectively, but 30.77 and 40.44 pmol/min/mg with *sn*-2,3-DAG excess, yielding 1.6-fold and 2.0-fold increases, respectively.

These results demonstrate that mammalian DGAT1 and DGAT2 enzymes can utilize both DAG enantiomers, with a notable preference for *sn*-2,3-DAG under these assay conditions. Unlike most plant DGAT1s, which exhibit strong selectivity for *sn*-1,2-DAG, both mammalian isoforms display broad enantiomer selectivity. This expanded substrate flexibility may reflect evolutionary adaptations that support isoform- and tissue-specific utilization of DAG enantiomers during TAG biosynthesis in mammals.

### Distinct DAG enantiomer selectivity of DGAT1 and DGAT2 from oleaginous microbes

To investigate DAG enantiomer utilization in microbial systems, we evaluated DGAT1- and DGAT2-like enzymes from the oleaginous yeast *Y. lipolytica* (Yl) and the marine microalga *N. oceanica* (No), both known for their industrial relevance in lipid production [38,39]. In *Yarrowia*, the DGAT1 and DGAT2 orthologs are designated as DGA2 and DGA1 [40], respectively, while in *Nannochloropsis*, the DGAT2 ortholog examined here corresponds to DGTT5 [41], which is highly expressed compared to other isoforms. These assignments are based on their sequence clustering with respective DGAT1 or DGAT2 homologs from other species (Figure 1). Each DGAT gene was expressed in yeast and the microsomal DGAT assays were performed as above.

**Figure 5:**
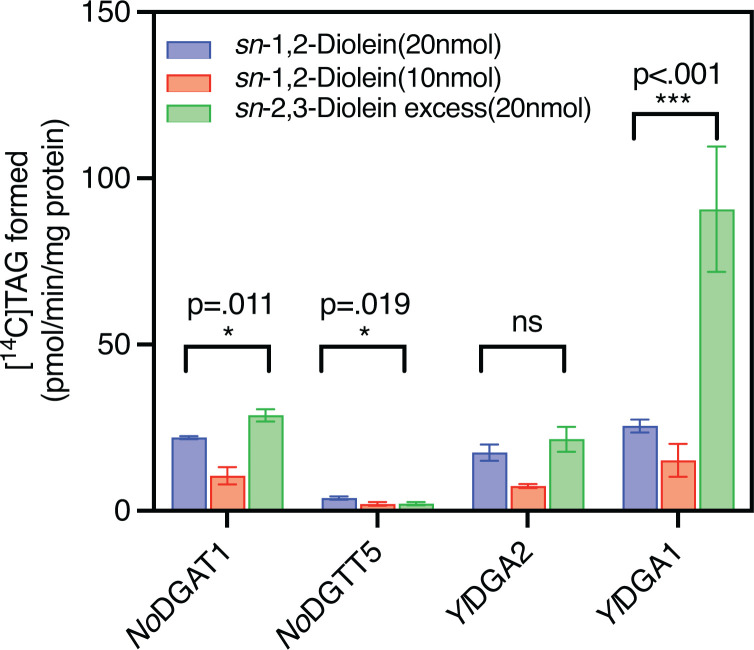
Distinct DAG enantiomer selectivity of DGAT1 and DGAT2 homolog proteins from Oleaginous Microbes, *N. oceanica* and *Y. lipolytica (Yl)* Enzyme assays were performed using [^14^C]18:1-CoA (1 nmol) as the acyl donor and either *sn*-1,2-diolein at 20 nmol (blue) or 10 nmol (red), or 20 nmol *sn*-2,3-diolein excess (green; ∼89% *sn*-2,3-diolein, ∼8% *sn*-1,2-diolein and ∼2.8% of *sn*-1,3-diolein) as acyl acceptors. Bars represent the mean ± SD of three technical replicates. Statistical significance was determined by one-way ANOVA followed by post-hoc tukey’s using GraphPad Prism. ns, not significant.

Among the *N. oceanica* enzymes, *No*DGAT1 produced 22.01 pmol/min/mg TAG with *sn*-1,2-DAG and 28.73 pmol/min/mg with *sn*-2,3-DAG, a 1.3-fold increase that reflects a slight but significant preference for the *sn*-2,3 isomer (Figure 5). *No*DGTT5 showed the inverse relationship, with TAG production decreasing from 3.83 pmol/min/mg with *sn*-1,2-DAG to 2.16 pmol/min/mg with *sn*-2,3-DAG, indicating a 1.8-fold preference for *sn*-1,2-DAG. In *Y. lipolytica, Yl*DGA2 generated 17.53 pmol/min/mg TAG with *sn*-1,2-DAG and 21.51 pmol/min/mg with *sn*-2,3-DAG (a 1.2-fold increase), but the difference was not statistically significant, indicating no real enantiomer selectivity. In contrast, *Yl*DGA1 exhibited a pronounced 3.6-fold increase in activity with *sn*-2,3-DAG (90.70 pmol/min/mg) compared to *sn*-1,2-DAG (25.51 pmol/min/mg), demonstrating strong, significant preference for the *sn*-2,3 isomer (Figure 5). Together, these results indicate that *No*DGAT1 modestly favors *sn*-2,3-DAG, while *No*DGTT5 favors *sn*-1,2-DAG. In contrast to the non-selective *Yl*DGA2, *Yl*DGA1 demonstrates strong selectivity for *sn*-2,3-DAG. This highlights species-specific strategies for DAG isomer utilization among microbial DGAT enzymes, likely shaped in part by distinct lipid metabolic architectures and ecological niches.

### DAG Acyl composition influences DGAT activity without altering enantiomer selectivity in camelina

**Figure 6:**
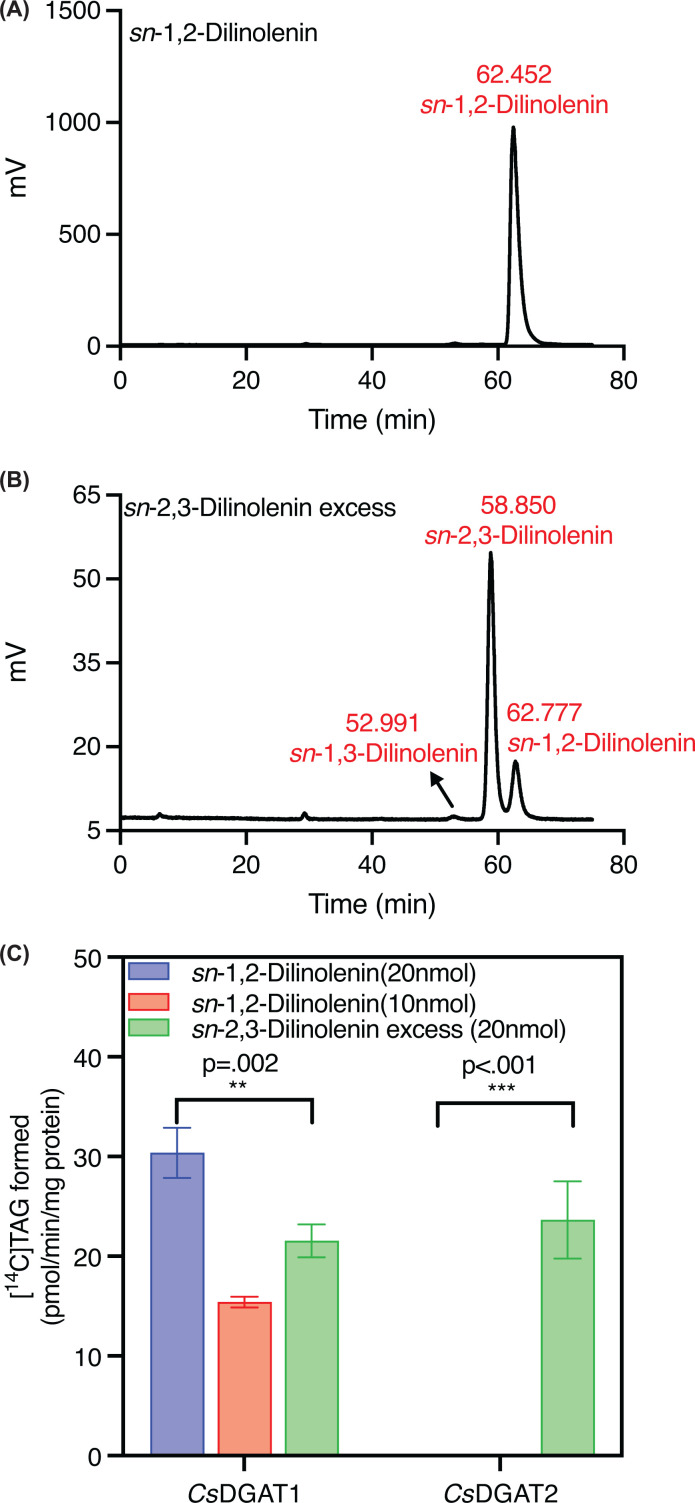
*Camelina sativa* DGAT1 and DGAT2 DAG enantiomer selectivity is retained with polyunsaturated DAG molecular species (**A,B**) HPLC chromatograms showing the separation of dilinolenin enantiomers. (**A**) *sn*-1,2-dilinolenin derived from *sn*-1,2-18:3-PC by phospholipase C digestion showed a single peak, confirming 100% *sn*-1,2 identity. (**B**) *sn*-2,3-dilinolenin excess generated from trilinolenin hydrolysis by *Rhizopus mehei* lipase that contain a mixture of ∼89% *sn*-2,3-dilinolenin, ∼8% sn-1,2-dilinolenin and ∼2.8% of sn-1,3-dilinolenin. (**C**) Enzyme assays were performed using [^14^C]18:1-CoA (1 nmol) as the acyl donor and either *sn*-1,2-dilinolenin at 20 nmol (blue) or 10 nmol (red), or 20 nmol *sn*-2,3-dilinolenin excess (green) as acyl acceptors. Bars represent the mean ± SD of three technical replicates. Statistical significance was determined by one-way ANOVA followed by post-hoc tukey’s using GraphPad Prism. ns, not significant.

To assess whether the acyl composition of DAG affects DGAT enantiomer selectivity, we used *C. sativa* as a representative species and evaluated the activity of its DGAT1 and DGAT2 isoforms with DAG substrates containing the omega-3 FA linolenic acid (18:3) (Figure 6). *Camelina* naturally accumulates high levels of 18:3 PUFAs in its seed oil and is frequently used as a platform for metabolic engineering of long-chain omega-3 FAs such as eicosapentaenoic acid (EPA) and docosahexaenoic acid (DHA) [21,42]. Thus, 18:3-DAG represents a metabolically relevant molecular species for this oilseed, making it a suitable model for testing how acyl chain composition may influence DGAT activity and enantiomer utilization.

HPLC analysis of dilinolenin enantiomers displayed a pattern similar to that observed for diolein enantiomers. The unlabeled *sn*-1,2-dilinolenin produced a single sharp peak, confirming 100% purity of the *sn*-1,2 isomer (Figure 6A). In contrast, the racemic dilinolenin generated from [^12^C]trilinolenin via partial hydrolysis with *Rhizomucor miehei* TAG lipase consisted of approximately 79% *sn*-2,3- dilinolenin, 20.6% *sn*-1,2- dilinolenin, and 1.2% *sn*-1,3- dilinolenin based on peak integration (Figure 6B) Accordingly, this preparation was designated as ‘*sn*-2,3- dilinolenin excess’.

Microsomal DGAT assays were performed using [^14^C]18:1-CoA as the acyl donor and either *sn*-1,2-dilinolenin (10 or 20 nmol) or *sn*-2,3- dilinolenin excess substrate (20 nmol) as the acyl acceptor. *Cs*DGAT1 exhibited strong activity with *sn*-1,2-dilinolenin, producing 15.41 pmol min^−1^ mg^−1^ at 10 nmol and 30.37 pmol min^−1^ mg^−1^ at 20 nmol, indicating higher TAG accumulation at the increased substrate concentration tested (Figure 6C). With *sn*-2,3- dilinolenin excess, *Cs*DGAT1 produced 21.56 pmol min^−1^ mg^−1^ TAG, significantly above the expected contribution from the *sn*-1,2 fraction (∼6.3 pmol min^−1^ mg^−1^, based on 20.6% content) (Figure 6C). This demonstrates that *Cs*DGAT1 can acylate both DAG enantiomers, though it retains an ∼1.4-fold preference for *sn*-1,2-dilinolenin. In contrast, *Cs*DGAT2 was completely inactive with *sn*-1,2-dilinolenin (0 pmol min^−1^ mg^−1^) but displayed strong activity with *sn*-2,3-DAG excess substrate (23.65 pmol min^−1^ mg^−1^) (Figure 6C). This pattern mirrors results obtained with diolein enantiomers as mentioned above. The consistent inactivity toward *sn*-1,2-DAG and strong TAG synthesis with *sn*-2,3-DAG confirm that *Cs*DGAT2 is highly selective for the *sn*-2,3 enantiomer, and that its stereochemical preference is unaffected by DAG acyl chain composition.

When compared to the diolein data (Figure 2), *Cs*DGAT1 displayed slightly reduced overall activity with dilinolenin, but the relative pattern of enantiomer utilization remained similar. *Cs*DGAT2, on the other hand, showed even greater activity with racemic dilinolenin than with diolein, suggesting that DAG acyl composition may enhance its catalytic efficiency when the correct enantiomer is present.

### Differential DAG and Acyl-CoA specificities of arabidopsis DGAT isoforms explain limited HFA incorporation in engineered plants

**Figure 7:**
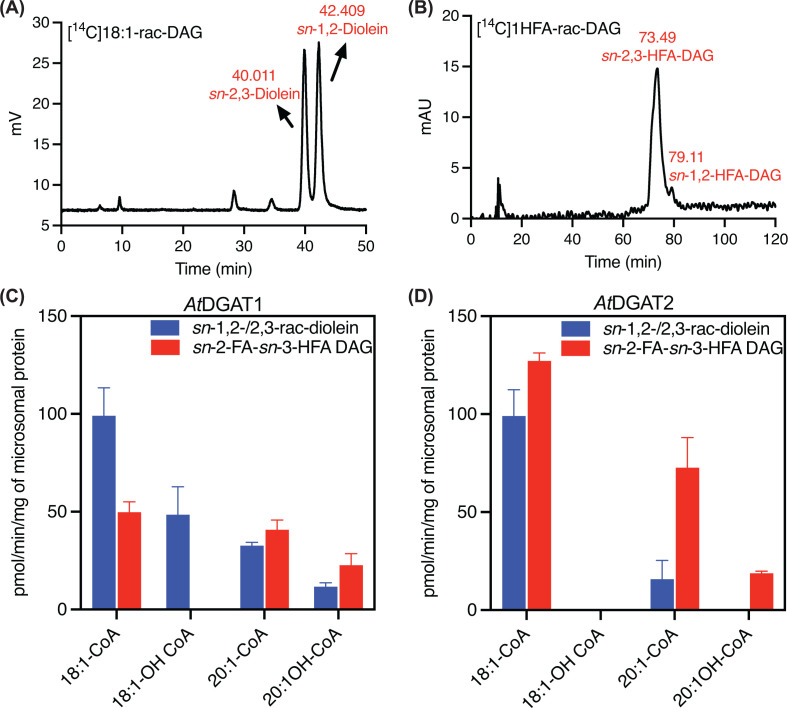
*At*DGAT1 and *At*DGAT2 activities with different acyl-CoAs using 18:1-rac-DAG and 1HFA-rac-DAG substrates (**A,B**) HPLC chromatograms showing DAG enantiomer composition of the DAG substrates used. (**A**) [^14^C]18:1-rac-DAG contained approximately equal proportions of *sn*-1,2-diolein and *sn*-2,3-diolein. (**B**) [^14^C]1HFA-DAG (mainly *sn*-2-FA-*sn*-3-HFA-DAG) generated from [^14^C]2HFA-TAG (*sn*-1,3-HFA-*sn*-2-FA-TAG; isolated from *P. fendleri* and enriched in 20:1OH) by *Rhizomucor miehei* lipase digestion contained ∼90% *sn*-2-FA-*sn*-3-HFA-DAG and ∼10% *sn*-1-HFA-*sn*-2-FA-DAG. (**C,D**) Activities of AtDGAT1 and AtDGAT2 with 20 nmol of 18:1-rac-DAG or 1HFA-rac-DAG and 5 nmol of the indicated acyl-CoAs. Activities were assayed using microsomes prepared from yeast expressing each DGAT isoform. Bars represent mean ± SD of three technical replicates.

Arabidopsis is frequently utilized as a proof-of-concept species for engineering unusual FA accumulation. However, prior works indicated that Arabidopsis TAG synthesis does not efficiently utilize unusual FAs as efficiently as the TAG synthesizing enzymes from species that naturally accumulate the unusual FAs [5,23,43]. However, recently while characterizing the enzymes involved in TAG remodeling from *P. fendleri* (*Pf*), singular overexpression of the TAG lipase *Pf*TAGL1 in an Arabidopsis background previously engineered to produce HFA led to increases in both total oil and HFA content [19]. Therefore, we postulated if the product of the *Pf*TAGL1, *sn*-2,3-DAGs that contain HFA, may have been a better substrate for *At*DGAT1 or *At*DGAT2 than the PC-derived DAGs utilized *in vivo*. Therefore, to assess how DAG acyl and stereochemistry and acyl-CoA structure influence Arabidopsis DGAT isoform activity, microsomal DGAT assays were conducted using two distinct DAG substrates, [^14^C]-labeled *sn*-1,2/2,3-rac-diolein (18:1-rac-DAG), which contains an approximately equal mixture of *sn*-1,2- and *sn*-2,3-diolein (Figure 7A), and ^14^C-labeled 1HFA-*rac*-DAG, which is highly enriched (∼90%) in *sn*-2-FA-*sn*-3-HFA DAG and 10% of *sn*-1-HFA-*sn*-2-FA-DAG (Figure 7B) (FA indicates 18:1, 18:2, or 18:3 and HFA is lesquerolic acid, 20:1-OH; our HPLC analysis indicated 18:3/HFA-DAG as the major species, Supplementary Figure S3). Accordingly, ^14^C-labeled 1HFA-*rac*-DAG was designated as ‘*sn*-2-FA-*sn*-3-HFA DAG excess’. These DAG substrates were assayed with four acyl donors: 18:1-CoA, 20:1-CoA, 18:1-OH-CoA and 20:1-OH-CoA. These substrate combinations were selected to systematically evaluate whether DAG enantiomer/acyl composition and acyl-CoA modifications (chain length and hydroxylation) alter *At*DGAT1 and *At*DGAT2 selectivity, providing insight into their potential roles in engineering TAG remodeling pathways to enhance unusual FAs.

*At*DGAT1 displayed broad tolerance for DAG enantiomers (Figures 2 and 7), but its activity varied depending on both DAG stereochemistry/acyl composition and acyl-CoA species. With 18:1-CoA, *At*DGAT1 produced 49.7 ± 5.3 pmol/min/mg TAG from *sn*-2-FA-*sn*-3-HFA excess DAG, which was roughly half the amount observed with *sn*-1,2/2,3-rac-diolein (99.1 ± 14.2 pmol/min/mg). With 20:1-CoA, TAG accumulation from *sn*-2-FA-*sn*-3-HFA excess DAG (40.7 ± 5.0 pmol/min/mg) was modestly higher than from *sn*-1,2/2,3-rac-diolein (32.7 ± 1.7 pmol/min/mg). *At*DGAT1 retained measurable activity with 20:1-OH-CoA on *sn*-2-FA-*sn*-3-HFA excess DAG (22.7 ± 5.9 pmol/min/mg), double that of TAG levels formed from *sn*-1,2/2,3-rac-diolein with the same donor (11.6 ± 2.1 pmol/min/mg). In contrast, no TAG formation was observed with 18:1-OH-CoA and *sn*-2-FA-*sn*-3-HFA enriched DAG, while *sn*-1,2/2,3-*rac*-diolein produced 48.5 ± 14.3 pmol/min/mg TAG when paired with the same acyl-CoA substrate. Notably *At*DGAT1 only produced a TAG molecular species containing both *sn*-1/*sn*-3 HFA when utilizing *sn*-2-FA-*sn*-3-HFA enriched DAG and 20:1OH-CoA that may indicate higher flexibility for unusual FA containing substrates when the FAs are >18-carbons. The results of Figure 7C underscore that *At*DGAT1 can utilize both DAG enantiomers, but the utilization of unusual FA containing substrates varies greatly with DAG acyl composition and acyl-CoA compatibility. These results are consistent with our other enantiomer-specific assays showing that AtDGAT1 can acylate both DAG enantiomers when common FA substrates are used (e.g. both 18:1 and 20:1).

*At*DGAT2 exhibited a distinct preference for *sn*-2-FA-*sn*-3-HFA excess DAG, particularly when paired with very-long-chain acyl-CoAs. Thus, the inclusion of HFA-containing DAG did not alter the *sn*-2,3-DAG enantiomer selectivity of AtDGAT2 (Figures 2 and 7D). With 18:1-CoA, *At*DGAT2 produced 127.2 ± 4.0 pmol/min/mg TAG from *sn*-2-FA-*sn*-3-HFA enriched DAG, which was ∼1.3-fold higher than *sn*-1,2/2,3-rac-diolein (99.0 ± 13.4 pmol/min/mg). This preference became more pronounced with 20:1-CoA, where TAG accumulation from *sn*-2-FA-*sn*-3-HFA excess DAG (72.7 ± 15.4 pmol/min/mg) was ∼4.6-fold higher than *sn*-1,2/2,3-rac-diolein (15.8 ± 9.6 pmol/min/mg), indicating enhanced utilization of *sn*-2,3-DAGs in combination with longer acyl chains. Hydroxylated acyl donors elicited a selective response: with 18:1-OH-CoA, *At*DGAT2 displayed no activity with both DAG substrates, whereas 20:1-OH-CoA supported modest TAG synthesis only with *sn*-2-FA-*sn*-3-HFA excess DAG (18.7 ± 1.1 pmol/min/mg) but not with *sn*-1,2/2,3-rac-diolein. Collectively, these findings demonstrate that *At*DGAT2 preferentially acylates *sn*-2,3-excess DAGs, especially when combined with longer-chain acyl donors, and selectively accommodates hydroxylated acyl-CoAs depending on DAG stereochemistry. Moreover, *At*DGAT2 can acylate *sn*-1,2/2,3-rac-diolein (a mixture of *sn*-1,2- and *sn*-2,3-DAG) only with common acyl-CoAs but not with hydroxylated acyl-CoAs, underscoring its constrained substrate compatibility.

These results provide critical insight into why Arabidopsis DGATs show limited efficiency in incorporating unusual FAs *in vivo* during HFA bioengineering attempts [43–46]. *At*DGAT1 functions as a more generalist enzyme capable of utilizing both DAG enantiomers but is less flexible to utilize unusual FA containing substrates, while *At*DGAT2 appears specialized for remodeling lipase-derived *sn*-2,3-DAG intermediates and is more selective for utilization of some (but not all) unusual FA containing acyl-CoA species.

## Discussion

### Distinct DAG enantiomer preferences of DGAT isoforms reflect evolutionary adaptations in TAG biosynthesis across the tree of life

Our comparative analysis across twelve evolutionarily diverse species reveals that DGAT enzymes from plants, mammals and microbes have evolved with isoform and lineage-specific preferences for DAG enantiomers, reflecting the underlying metabolic architecture of TAG biosynthesis in each species. This directly addresses our central question whether DAG enantiomer selectivity is a conserved mechanistic feature or an adaptive specialization tied to a specific metabolic context, such as TAG remodeling to accumulate lesquerolic acid in *P. fendleri* [19]. The DGAT1 enzymes from Brassicaceae crop species examined here (*Camelina* and pennycress) consistently preferred *sn*-1,2-DAG, whereas DGAT2 isoforms exhibited strong selectivity toward *sn*-2,3-DAG, revealing a clear functional division between isoforms. These findings might also help to explain the biochemical impetus behind the convergent evolution that drew these two enzyme types together [33]. This pattern suggests that DGAT1 primarily channels Kennedy pathway or PC-derived *sn*-1,2-DAG into TAG synthesis, while DGAT2 functions preferentially on TAG turnover-derived *sn*-2,3-DAG, consistent with a role in TAG remodeling similar to that described in the related Brassicaceae *P. fendleri* [5,19]. Interestingly, DGAT1 from the model species Arabidopsis had little selectivity for DAG enantiomers (Figure 2) indicating an evolved difference from the other Brassicaceae members. Additionally, it is a reminder to be cautious when using results derived from model species to those predicted in crops, which may possess significant species-specific differences in lipid metabolism.

DGAT1 enzymes from non-Brassicaceae oil crops (peanut, cotton, tung, and castor) maintained a strong and conserved preference for *sn*-1,2-DAG, suggesting that this enantiomeric bias is an ancestral feature of plant DGAT1s. However, DGAT2 enzymes displayed broader diversification, with some species (e.g. peanut and tung) displaying *sn*-2,3 DAG selectivity, while others (e.g. castor) favored *sn*-1,2-DAG or were nearly non-selective (e.g. cotton). This diversity likely reflects species-specific adaptation to mechanisms of DAG pool production, DAG composition, and acyl fluxes, particularly in plants that accumulate unusual FAs where different lipid biosynthetic pathways are used to sequester the unusual FAs in TAG and not in membrane lipids. For example, *P. fendleri* utilizes PC-derived *sn*-1,2-DAG (that does not contain lesquerolic acid) for initial TAG synthesis with *sn*-3 lesquerolic acid, followed by TAG remodeling lipase activity and the action of *sn*-2,3-DAG selective *Pfe*DGAT2 to incorporate lesquerolic acid into the *sn*-1 position of TAG [19,32]. Thus, TAG remodeling (including enantiomer selective DGATs) is used to limit the flux of unusual FAs through PC (where it can affect membrane structure/function) for eventual PC-derived DAG and TAG synthesis. However, castor takes a different strategy to accumulate ricinoleate. All three castor endosperm Kennedy pathway acyltransferases effectively utilize ricinoleoyl-CoA to produce *de novo sn*-1,2-DAG and, ultimately, TAG containing three ricinoleate groups [5,27]. Here we demonstrate that castor DGAT1 and DGAT2 both prefer *sn*-1,2-DAG consistent with the endogenous pathway of castor TAG biosynthesis that does not utilize a membrane lipid as an intermediate to TAG biosynthesis and thus does not need TAG remodeling to incorporate the unusual FA after formation of the membrane lipid intermediate.

Both human and mouse DGAT1 and DGAT2 isoforms utilized both DAG enantiomers efficiently, but with slightly higher selectivity for *sn*-2,3-DAG. This broad stereochemical tolerance is fully consistent with both the physiology and evolutionary origin of mammalian TAG synthesis [24]. Mammals obtain most TAG carbon from dietary TAG, which is hydrolyzed in the intestine to *sn*-2-MAG plus NEFAs, then re-esterified via MGAT pathways. MGATs can acylate either the *sn*-1 or *sn*-2 position of MAG, generating both *sn*-1,2- and *sn*-2,3-DAG *in vivo* [9]. In addition, TAG lipolysis inside tissues also generates mixed DAG pools. Adipose TAG lipase (ATGL) initiates TAG hydrolysis in adipocytes, and in the presence of its co-activator comparative gene identification-58 (ABHD5), ATGL’s regioselectivity broadens: instead of producing only *sn*-1,2-DAG, CGI-58 activation enables hydrolysis at *sn*-1 (but not *sn*-3) to produce *sn*-2,3-DAG as well [20]. Thus, mammals inevitably generate substantial *sn*-2,3-DAG during both TAG digestion and lipolysis, and the DGAT1 and DGAT2 have evolved to productively utilize both enantiomers. The observed *sn*-2,3-DAG preference *in vitro* reflects this biology: *sn*-2-MAG is the dominant intestinal intermediate, and *sn*-2,3-DAG is a physiologically prominent substrate *in vivo*. Mammalian DGATs therefore appear adapted not to enforce stereochemical constraints, but rather to sustain high TAG flux across a system with continuous TAG synthesis, breakdown, and recycling.

At least one DGAT from both microorganisms evaluated also demonstrated capability to utilize *sn*-2,3-DAG, albeit with different DGATs. *Yarrowia* store TAG under excess carbon or specific nutrient imbalance and then mobilize it when nutrients become limiting or when growth resumes. This cyclical ‘store TAG under favorable conditions and burn TAG when conditions shift’ strategy is a core part of yeast lipid homeostasis and ecological fitness [47]. Under such a regime, *sn*-2,3-DAG might become a natural intermediate of TAG cycling. In Nannochloropsis, TAG is produced under limiting nutrient conditions and then remobilized for growth when nutrient conditions improve. Thus, the conditions are opposite but the biological significance may be similar to *Yarrowia* in that the ability to utilize *sn*-2,3-DAG allows dynamic TAG cycling to improve organismal fitness under different environmental and growth conditions. Collectively, these findings demonstrate that stereochemical selectivity of DGATs is a conserved yet evolutionarily adaptable trait, shaped by the source and dynamics of DAG pools within each organism. Species that accumulate TAG predominantly through *de novo* biosynthesis may tend to favor *sn*-1,2-DAG–utilizing DGATs, while those with active TAG turnover and resynthesis or TAG remodeling pathways might have evolved enzymes capable to use or specialized for *sn*-2,3-DAG utilization. While consistent patterns were observed among the plant DGATs examined here, the limited number of mammalian and microbial DGATs analyzed suggests that broader conclusions across kingdoms will require characterization of additional DGAT isoforms from diverse species.

### Differential DAG enantiomer selectivity implies additional specialization of plant TAG synthesis between DGAT1 and DGAT2 to control TAG *sn*-3/*sn*-1 fatty acid composition

DGATs have been highly characterized for their acyl-CoA substrate FA selectivity [22]. More recently, the selectivity for the combined acyl-CoA and DAG molecular species has revealed distinct differences between DGAT1 and DGAT2 from *C. sativa*. *Cs*DGAT1 activity was highest when the DAG contained 18:1 or 16:0, but *Cs*DGAT2 activity was highest when both DAG and acyl-CoA contained PUFAs [21]. In addition to the molecular species selectivity, we demonstrate that *Cs*DGAT1 and *C*sDGAT2 have distinct preferences for *sn*-1,2-DAG and *sn*-2,3-DAG respectively, and this preference is independent of acyl composition (Figures 2 and 6). Similar DGAT1 and DGAT2 DAG enantiomer selectivity for *sn*-1,2-DAG and *sn*-2,3-DAG, respectively, was found for pennycress, peanut, and tung. Cotton DGAT1 was selective for *sn*-1,2-DAG while *Gh*DGAT2 utilized both enantiomers. Together these results suggest that DGAT1 and DGAT2 have evolved to not only control overall TAG FA composition, but to differentially control the composition at the TAG *sn*-3 and *sn*-1 positions, respectively. This result implies that if TAG remodeling is active in each of these species during seed oil accumulation, then the FA composition of the TAG *sn*-1 position may not be completely controlled by GPAT9 activity [48], but also by DGAT2 activity utilizing TAG-derived *sn*-2,3-DAG. This conclusion has direct implications for breeding or engineering of plant seed oil FA compositions where the goal is to control the compositions of all three positions of TAG.

Likewise, the combined enantiomer and substrate molecular species selectivity of Arabidopsis DGAT1 and DGAT2 for unusual hydroxylated FAs helps to explain the bottlenecks in engineering Arabidopsis to accumulate HFA in TAG. Expression of the castor RcFAH12 hydroxylase alone in Arabidopsis produces less than 17% of HFA in TAG, and very little of the TAG molecular species have more than one HFA unless castor acyltransferases are also expressed [49,50]. Both AtDGAT1 and AtDGAT2 readily acylated both common FA DAG (*sn*-1,2/2,3 diolein) and *sn*-2,3-1HFA-DAG substrates using 18:1-CoA or 20:1-CoA. However, when the DAG acceptor contained a *sn*-2,3-HFA-DAG, both enzymes failed to produce TAG with 18-carbon HFA-CoA donors. This suggest that the bottleneck is not a generalized inability to use *sn*-2,3 DAG, but it is specifically the *combination* of *sn*-2,3-HFA-DAG and 18-carbon HFA-CoA as an acyl acceptor/donor pair. Importantly, when the donor was switched to 20-carbon HFA-CoA, TAG formation was partially restored for both DGATs, revealing that chain-length affected the enzymatic activity toward acyl-CoA species containing hydroxylated FAs. AtDGAT2 especially showed strong *sn*-2,3-DAG utilization when paired with common and longer chain acyl-CoA donors. These assays suggest a mechanistic missing link: Arabidopsis DGAT1 and DGAT2 are not inherently unable to use *sn*-2,3-DAG, but they are inefficient at specifically esterifying a 18-carbon HFA-CoA onto *sn*-2,3-HFA-DAG intermediates. Thus, even if some TAG remodeling to produce s*n*-2,3-DAG is occurring in Arabidopsis the combined enantiomer and substrate molecular species selectivity suggests Arabidopsis DGATs are not efficient for accumulation of TAG with multiple HFA. Similar enantiomer and substrate molecular species selectivities likely occur in other species chosen for bioengineering of different FA compositions [23], and thus support the need to engineer both the robust production of the FA of choice and to replace the endogenous DGATs with enzymes containing both the molecular species and enantiomer selectivities needed to accumulate the FA of choice at all three positions of the DAG backbone [51].

### Plant DGAT enantiomer preferences imply TAG remodeling may be more widespread than currently recognized

The highly dynamic nature of TAG metabolism in mammals and microorganisms logically correlates with the idea that DGATs should be able to utilize *sn*-2,3-DAG to resynthesize TAG. However, the majority of plant TAG production is controlled developmentally, with TAG accumulation during seed development and complete TAG degradation during seed germination. Despite the mass accumulation of TAG during seed development, recent discovery of TAG remodeling in *Physaria fendlei* indicates that TAG metabolism can involve cycles of anabolic and catabolic reactions within a shared biosynthetic pathway. Key to *Physaria* TAG remodeling for lesquerolic acid accumulation is the ability of *Pfe*DGAT2 to utilize TAG lipase (*Pfe*TAGL1) generated *sn*-2,3-TAG to incorporate lesquerolic acid at the *sn*-1 position. Thus, the capacity of a plant DGAT to efficiently utilize *sn*-2,3-TAG indicates the presence of one of the key enzymatic requirements for TAG remodeling in that species. Of the DGAT1s and DGAT2s from eight plant species analyzed here, all except castor contained at least one DGAT that was selective for *sn*-2,3-DAG or a DGAT that would utilize either enantiomer equally. Additionally, the possibility for TAG remodeling may be supported by the changing composition of TAG during seed development. In Arabidopsis, Camelina, and pennycress, early seed TAG is enriched in 16:0, 18:0, and 18:1/18:2, but during seed maturation the TAG pool progressively shifts toward higher 18:3 and ≥20-carbon monounsaturated FAs on a mass basis [52–55]. Thus, combined with the capacity to utilize *sn*-2,3-DAG this temporal shift in TAG composition may imply that TAG remodeling contributes to final seed oil composition.

Together, these data strongly suggest that TAG remodeling is almost certainly not unique to *Physaria*. Rather, enantiomer selective utilization of *sn*-2,3-DAG appears to be a generalizable feature of oilseed metabolism, particularly in many species that accumulate high-value unusual FAs, PUFAs, or VLCFAs in mature seed TAG. However, the degree to which each lineage actually uses remodeling remains unknown and needs to be tested with long-term pulse-chase isotopic tracing, similar to the original *Physaria* flux experiments [32]. Further, the extent of remodeling is likely not determined solely by DGAT identity and selectivity, but also by the presence of acyl selective TAG lipases, and the developmental timing of DAG and TAG molecular species dynamics. In addition, interpretation of DAG enantiomer selectivity from mixed DAG substrate preparations should be approached cautiously. Previous work with Arabidopsis DGATs showed that addition of *sn*-2,3-DAG reduced AtDGAT1 activity toward *sn*-1,2-DAG by approximately 50%, suggesting that the nonpreferred DAG enantiomer may influence enzyme activity through substrate competition or inhibitory effects. However, the response was not linear with increasing *sn*-2,3-DAG concentration, and the *sn*-2,3-DAG preparation still contained residual *sn*-1,2-DAG, making the mechanism difficult to resolve [56]. Because complete purification of DAG enantiomers in sufficient quantities for enzymatic assays remains technically challenging, residual contamination of the alternate enantiomer may influence apparent selectivity measurements. Nevertheless, the strong and consistent selectivity patterns observed here across multiple DGAT isoforms clearly support intrinsic stereochemical preferences between DGAT1 and DGAT2 enzymes. Recent cryo-EM structures and advances in AlphaFold-based protein structure prediction provide an important framework for future studies aimed at identifying the structural determinants of DAG enantiomer selectivity among DGAT isoforms. Comparative structural analyses between DGAT1 and DGAT2 enzymes with contrasting stereochemical preferences may help define the molecular basis for substrate recognition and catalytic specialization. Addtionally, whether other TAG synthesizing enzymes such as phospholipid:diacylglycerol acyltransferases [5] also have DAG enantiomer selectivity and contribute to TAG remodeling remains to be evaluated. This work therefore suggests a new conceptual framework: TAG remodeling is likely a widespread and underappreciated layer of plant TAG assembly, and mapping lineage-specific remodeling dynamics will be critical for both for understanding lipid metabolism and for engineering designer oils in plants, or lipid accumulating microorganisms.

## Methods

### Chemicals and substrates

Unless otherwise noted, all chemicals, solvents, and reagents were obtained from Fisher Scientific, USA. [^14^C]oleoyl-CoA (18:1-CoA) and [^14^C]*sn*-1,2/2,3-rac-diolein was purchased from American Radiolabeled Chemicals, Inc. (USA). *sn*-1,2-diolein and 1, 2-dilinolenoyl-sn-glycero-3-phosphocholine (*sn*-1,2-18:3-PC) was obtained from Avanti Polar Lipids (USA). Racemic mixtures of [^12^C]*sn*-1,2/2,3-diolein and [^12^C]*sn*-1,2/2,3-diolinolenin were generated from [^12^C]triolein and [^12^C]trilinolenin respectively, via partial hydrolysis using TAG lipase from *Rhizomucor miehei* (Sigma–Aldrich). [^14^C]1HFA-*rac*-DAG was generated from [^14^C]2HFA-TAG as described previously [19]. *Sn*-1,2-dilinolenin was prepared from hydrolysis of *sn*-1,2-18:3-PC by using phospholipase C from *Clostridium perfringens* (Sigma–aldrich). The resulting DAG mixtures were separated by thin-layer chromatography (TLC) using a solvent system of hexane:diethyl ether:acetic acid (70:30:1, v/v/v). The band corresponding to the *sn*-1,2/2,3-*rac*-DAG fraction was scraped and eluted according to previously described protocols [57]. Unlabeled oleoyl-CoA (18:1-CoA), ricinoleoyl-CoA (18:1-OH-CoA), 11-eicosenoyl-CoA (20:1-CoA), and lesqueroyl-CoA (20:1-OH-CoA) were chemically synthesized from free FAs and CoA as described previously [58].

### *In silico* analysis

Phylogenetic tree of species in various taxa was constructed using NCBI taxonomy browser (https://www.ncbi.nlm.nih.gov/Taxonomy/CommonTree/wwwcmt.cgi) and Interactive tree of life (iTOL) tool (https://itol.embl.de/) [59]. Sequence identity and similarity was analyzed by using NCBI protein blast (BLASTp) with *Pfe*DGAT1 and *Pfe*DGAT2 protein sequences as queries and all other species homolog DGAT1 and DGAT2 sequences, respectively as subjects. MSA was created using Clustal Omega (https://www.ebi.ac.uk/jdispatcher/msa/clustalo).

### High-performance liquid chromatography analysis of DAG enantiomers

To assess the enantiomeric composition of the isolated DAGs, HPLC was performed with slight modifications to established procedures [60]. Briefly, a 10-μl aliquot of sample (containing 10–50 μg of DAGs in n-hexane) was injected into an Agilent 1260 Infinity II HPLC system equipped with two columns connected in series: a normal-phase silica column (Hypersil GOLD™ Silica, 5 μm, 4.6 × 150 mm, Thermo Scientific, USA) and a chiral column (CHIRALCEL^®^ OD-H, cellulose tris(3,5-dimethylphenylcarbamate)-coated silica, 5 μm, 4.6 × 250 mm, Daicel Chemical Industries, Japan). The mobile phase consisted of n-hexane and 2-propanol (97.7:2.3, v/v) delivered at 0.5 ml/min at 10°C. Eluted peaks were monitored using both a diode array detector set at 210 nm and an evaporative light scattering detector (ELSD; Agilent 1260 Infinity II ELSD) with the following settings: evaporator temperature 50°C, nebulizer temperature 25°C, gas flow rate 1.4 SLM, LED power at 100%, sampling rate 80 Hz, smoothing 30, and PMT gain 1. Peak areas were integrated, and the enantiomeric composition was calculated as the ratio of the area of each DAG peak to the sum of all DAG peak areas.

### Gene cloning, yeast transformation and microsomal preparations

Codon-optimized or native DGAT1 and DGAT2 genes from various species (see Supplementary Table S1) were individually cloned into the yeast expression vector pYES2/NT-C (Invitrogen, USA), which carries a GAL1 promoter and URA3 marker gene for selection. The recombinant plasmids were introduced into the TAG-deficient *Saccharomyces cerevisiae* strain H1246 [36] using the lithium acetate transformation method as described in Invitrogen manual. H1246 cells transformed with the empty pYES2/NT-C vector were used as the vector control. Transformed cells were plated on synthetic minimal media lacking uracil supplemented with 2% glucose, following protocols outlined in the Invitrogen manual (Cat. No: V8252-20).

Recombinant colonies were cultured overnight at 30°C in synthetic media without uracil + 2% glucose, and gene expression was induced by transferring cells to SM-URA medium containing 2% galactose and 1% raffinose for 36 hours at 30°C [61]. To assess DGAT enzymatic activity and TAG biosynthesis, total lipids were extracted from the induced cultures, and TAG formation were analyzed by neutral lipid profiling, as previously described [61]. Microsomal membranes were isolated according to established protocols [62]. The protein concentration of microsomal fractions was quantified using the Pierce BCA Protein Assay Kit (Thermo Fisher Scientific, USA) with bovine serum albumin (BSA) as a standard.

### *In vitro* enzymatic assays

DGAT enzymatic activity was assessed in 100 μl reaction mixtures containing 50 mM HEPES buffer (pH 7.2) and 5 mM MgCl_2_ [21]. For enantiomer-specificity assays, the reactions included 1 nmol of [^14^C]-oleoyl-CoA (18:1-CoA), solubilized in FA–free bovine serum albumin (1 mg/ml), as the acyl donor and either diolein or dilinolenin as acyl acceptors. Concentrations of DAG added to the reaction includes either 20 nmol or 10 nmol of *sn*-1,2-DAG, or 20 nmol of *sn*-2,3-DAG excess dissolved in dimethyl sulfoxide. For Arabidopsis DGATs substrate FA composition specificity assays, five nmol of acyl-CoA and 20 nmol of [^14^C]*sn*-1,2/2,3-*rac*-diolein or [^14^C]1HFA-*rac*-DAG were used. Control reactions lacking added DAG were included to account for background DAG levels from the microsomal membranes and is subtracted from all other reactions during analysis. Microsomal membrane preparations equivalent to 50 μg of total protein were added to each reaction. The reactions were incubated at 30°C with shaking at 1250 rpm for 20 minutes and terminated by the addition of 120 μl of 150 mM acetic acid followed by 500 μl of chloroform: methanol (1:1, v/v). The lower organic phase containing lipids was collected, and one-fifth of the total lipid extract was used to measure total radioactivity using a PerkinElmer TriCarb 4910 liquid scintillation counter. The remaining lipid extract was evaporated under nitrogen, resuspended in 40 μl of chloroform, and applied to TLC plates (Analtech Silica gel HL; 20 cm × 20 cm; 250 μm thickness; 15 μm particle size) for separation of neutral lipids. The TLC was developed using a solvent system of hexane:diethyl ether:acetic acid (70:30:1, v/v/v). The developed TLC plates were exposed to multipurpose standard storage phosphor screens (GE healthcare, USA) in autoradiography cassettes for 24 hours. Radiolabeled lipid species were visualized using a Typhoon FLA 7000 phosphor imager, and TAG bands were quantified using ImageQuant TL 7.0 software (GE Healthcare, USA). TAG accumulation was calculated based on the proportion of radioactivity in the TAG band relative to the total radioactivity determined by scintillation counting.

### Statistical analysis

All data are presented as mean ± SD of triplicate assays. one-way ANOVA followed by post-hoc tukey’s statistical analyses were performed using GraphPad Prism version 10.1.2 to assess differences between groups.

## Supplementary Material

Supplementary Figures S1-S3

Supplementary Table S1

## Data Availability

All data are present in the article or supporting information.

## Abbreviations

ATGL: adipose TAG lipase
DAG: diacylglycerol
DGAT: diacylglycerol acyltransferase
ER: endoplasmic reticulum
FA: fatty acid
HFA: hydroxy fatty acid
HPLC: high-performance liquid chromatography
MAG: monoacylglycerol
MSA: multiple sequence alignment
PA: phosphatidic acid
PC: phosphatidylcholine
PUFA: polyunsaturated fatty acid
TAG: triacylglycerol
TLC: thin-layer chromatography
