# Supplementary material for: Diacylglycerol enantiomer selectivity of diacylglycerol acyltransferases highlights metabolic specialization in triacylglycerol synthesis across the tree of life
Source: Biosci Rep. 2026 Jun 22;46(7):BSR20260190. doi: 10.1042/BSR20260190 (PMC13286882; doi:10.1042/BSR20260190)

Supplementary figure S1: Multiple sequence alignment of DGAT1 sequences from species investigated in this study. For species names, see Supplementary data file S1.

```
YlDGAT1         ----------------------------MEVRRRKIDVLKAQKNGYESGPPSRQSSQ---29
HsDGAT1         -----------------------M-GDRGSSRRRRTGS-RPSSHGGG-GPA---------25
MmDGAT1         --------------------MGDR-GGAGSSRRRRTGS-RVSVQGGS-GPK---------28
NoDGAT1         ------------------------------------------------------------0
PfeDGAT1        MSILDSGGVS--TTDT------EYDGG-DKLRRRKSKS-DSTGLLSDS------------38
TaDGAT1         MAILDSGG----VTMPTENGGGEFADL-DTLRRRKSRS-DSNEPLSDSAPGTDAFPSDDV54
AtDGAT1         MAILDSAG----VTTVTENGGGEFVDL-DRLRRRKSRS-DSSNGLLLS-GSDNNSPSDDV53
CsDGAT1         MAILDSGGGGVSTATATENGGGEFVDL-RR---RKSRS-DSN-GVL-C--GSDNPPSDDV51
AhDGAT1a        MAIYQSVG----ATATVQGDS----GANSSTVRRRRGG-GATSATPL--E-KVE--LDAF46
GhDGAT1         MAMFESPEISGSSTATVIGTSRSESDLNHFAPRRRAVN-NAVDAGTRVVER---------50
VfDGAT1         MTIPETPDNSTDATT--SGGAESSSDLNLSLRRRRTAS-NSDGAVAELASKIDE--LE--53
RcDGAT1         MTILETPETLGV------ISSSATSDLNLSLRRRRTSN-DSDGALADLASKFDD--DDDV51


YlDGAT1         -PSSRASSRTRNK----HSSSTLSLSGLTMKV--------QKKPAGPPANSKTP--FLHI74
HsDGAT1         ----AAEEEVRDAAAG----PDVGAAGDAPAPAP------NKDGDA--GVGSGHWEL---66
MmDGAT1         ----VEEDEVRDAAVS----PDLGAGGDAPAPAPAPAHTRDKDGRT--SVGDGYWDL---75
NoDGAT1         ------------------------------------------------------------0
PfeDGAT1        TVSDDVRDRIDSVANEDAQG----LSGDNGSDTEIREIRGARSGGGGRGNSNATY-ARPS93
TaDGAT1         GAPSDARDRIDSAVDDA-QG-TANLAGDNGGDTEIRETGGGGGGGEARGDADTRYTYRPS112
AtDGAT1         GAPADVRDRIDSVVNDDAQGTAN-LAGDNNGGGDNNGGGR--GGGEGRGNADATFTYRPS110
CsDGAT1         GAPADVRDRIDSVVNDDAQGTTANLAGDNEI-RETGGGGRGGGGEGGRGNAETTYTYRPS110
AhDGAT1a        EGESSSEELAKDSSSDDN---------NNGNGVDDAS--KGNRQQNAADFSAVNFDYRPS95
GhDGAT1         -NNSGNGET------------------VDARDRMESANFSRENVNENPTNSDTRFTYRPS91
VfDGAT1         -SDAGGGQVIKDPGAE-MD-----SGTLKSNGKDCGTVKDRIENRENRGGSDVKFTYRPS106
RcDGAT1         RSEDSAENIIEDPVAAVTE-----LATAKSNGKDCVANSNKDKIDSHGGSSDFKLAYRPS106


YlDGAT1         KPVHTCCSTSMLSRDYDGSNPSFKGFKNIGMIILIVGNLRLAFENYLKYGISNP--FFD-131
HsDGAT1         -R-CHRLQDSLFSSD-SGF-SNYRGILNWCVVMLILSNARLFLENLIKYGILVDPIQVVS122
MmDGAT1         -R-CHRLQDSLFSSD-SGF-SNYRGILNWCVVMLILSNARLFLENLIKYGILVDPIQVVS131
NoDGAT1         MSMHKLTRPSVLSIE--YPSRDYTGYLNLAMIILGVHFSHAVVDC-VSL--VWRYGVQL-54
PfeDGAT1        VPAHRKAKESPLSSD-AIFKQSHAGLFNLCVVVLVALNSRLIIENLMKYGWLIRTDFWF-151
TaDGAT1         VPAHRRARESPLSSD-AIFKQSHAGLFNLCVVVLIAVNSRLIIENLMKYGWLIRTDFWF-170
AtDGAT1         VPAHRRARESPLSSD-AIFKQSHAGLFNLCVVVLIAVNSRLIIENLMKYGWLIRTDFWF-168
CsDGAT1         VPAHRRARESPLSSD-AIFKQSHAGLFNLCVVVLIAVNSRLIIENLMKYGWLIRTDFWF-168
AhDGAT1a        FPAHRRIRDSPLSSG-NIFKQSHAGLFNLCIVVLVAVNSRLIIENLMKYGWLIKTDFWF-153
GhDGAT1         VPAHWRIKESPLSSD-NIFQQSHAGLFNLCVVVLVAVNSRLIIENLMKYGWLIRTGFWF-149
VfDGAT1         VPAHRALKESPLSSD-NIFKQSHAGLFNLCIVVLVAVNSRLIIENIMKYGWLIKTGFWF-164
RcDGAT1         VPAHRSLKESPLSSD-LIFKQSHAGLFNLCIVVLVAVNSRLIIENLMKYGWLIKTGFWF-164
                         * :*        .. *  *  :::*     :  .:  :.


YlDGAT1         -PKITPSEWQLSGLLIVVAYAHILMAYAIESAAKLLFLSSKHHYMAVGLLHTMNTLSSIS190
HsDGAT1         LFLKDPYSWPAPCLVIAA-NVFAVAAFQVEKRLAVGAL---TEQA-GLLLHVANLATILC177
MmDGAT1         LFLKDPYSWPAPCVIIAS-NIFVVAAFQIEKRLAVGAL---TEQM-GLLLHVVNLATIIC186
NoDGAT1         -PKHSLVEVPCLMCALSL-TINIFLAWFTEYLASRRFF---PSSMAVGVLHSLNCLWTLL109
PfeDGAT1        -SSTSLRDWPLFMCCLSL-SIFPLASFTVEKLVLQKLI---SEPV-VIILHIIITTTAVL205
TaDGAT1         -SSTSLRDWPLFMCCISL-SIFPLAAFTVEKLVLQKCI---SEPV-VIILHIIITMTEVL224
AtDGAT1         -SSRSLRDWPLFMCCISL-SIFPLAAFTVEKLVLQKYI---SEPV-VIFLHIIITMTEVL222
CsDGAT1         -SSRSLRDWPLFMCCLSL-SFFPLAAFTVEKLVLQKCI---SEPV-VIFLHIIITMTEVL222
AhDGAT1a        -SSTSLSDWPLFMCCLTL-LLFPVASFIVEKLAQHKYI---PEPV-VVILHIIITSTSLL207
GhDGAT1         -SSRSLRDWPLFMCCLSL-PIFPIAAFVVEKLLQQNQI---SERT-LILLHILISTLAVL203
VfDGAT1         -SSRSLRDWPLLMCCLTL-PIFSLAAYLVEKLACRKYI---SAPT-VVFLHILFSSTAVL218
RcDGAT1         -SSRSLRDWPLFMCCLSL-PVFPLAAYLVEKAAYRKYI---SPPI-VIFLHVIITSAAVL218
                       .       :       . ::  *       :          .**       :
```

```
YlDGAT1         LLSYVVYYYLPNPVAGT-IVEFVAVILSLKLASYALTNSDLRKAAIHAQKLDKTQDDNEK249
HsDGAT1         FPAAVVLLVESITPVGSLLALMAHTILFLKLFSYRDVNSWCRRARA--KAA---------226
MmDGAT1         FPAAVALLVESITPVGSVFALASYSIMFLKLYSYRDVNLWCRQRRVKAKAV---------237
NoDGAT1         YPCHVAWSRPDVPLHTF-LLLFWSVIAFLKLVSWSHTNWDLRHAFFSRRARKSQAHLPAA168
PfeDGAT1        YPVYVTLRCDSAFLSGV-TLMLLTCIVWLKLVSYAHTNYDIRTLANSADK----------254
TaDGAT1         YPVYVTLRCDSAFLSGV-TLMLLTCIVWLKLVSYAHTSYDIRTLANSADK----------273
AtDGAT1         YPVYVTLRCDSAFLSGV-TLMLLTCIVWLKLVSYAHTSYDIRSLANAADK----------271
CsDGAT1         YPVYVTLSCDSAFLSGV-TLMLLTCIVWLKLVSYAHTNYDIRTLANSADK----------271
AhDGAT1a        YPIFVILRSDSAFLSGV-TLMLFACIVWLKLVSYGHTNYDLRALAKSNEK----------256
GhDGAT1         YPVVVILRCDSAFLSGI-ALMLLACIVWLKLVSYAHTNSDMRSVAKLTEK----------252
VfDGAT1         YPVSVILSCESAVLSGV-ALMLFACIVWLKLVSYAHTNFDMRAIANSVDK----------267
RcDGAT1         YPASVILSCESAFLSGV-TLMELACMVWLKLVSYAHTNYDMRAIADTIHK----------267
                    *                    :  *** *:  ..   *

YlDGAT1         ESTSSSSSSDDAETLADIDVIPAYYAQLPYPQNVTLSNLLYFWFAPTLVYQPVYPKTERI309
HsDGAT1         ---S------A----GKKASSAAAPHTVSYPDNLTYRDLYYFLFAPTLCYELNFPRSPRI273
MmDGAT1         ---S------T----GKKVSGAAAQQAVSYPDNLTYRDLYYFIFAPTLCYELNFPRSPRI284
NoDGAT1         ALHE------D----GYNNAKPLESGATRYPHSVSLSNISFFFFCPTLCYQPDYPRAPTI218
PfeDGAT1        ---A------N----------------PEVSYDVSFKSLAYFMVAPTLCYQPSYPRSPCI289
TaDGAT1         ---A------N----------------PEVSYYVSLKSLAYFMVAPTLCYQLSYPRSPCI308
AtDGAT1         ---A------N----------------PEVSYYVSLKSLAYFMVAPTLCYQPSYPRSACI306
CsDGAT1         ---A------N----------------PEVSYYVSLKSLAYFMVAPTLCYQPSYPRSPCI306
AhDGAT1a        ---V------E----GL-----PSTLSMDYPYDVSIRRLAYFMLAPTLCYQPSYPRTPSI298
GhDGAT1         ---G------S----------------EGCMYNVSFRSLAYFMAAPTLCYQTSYPRTASI287
VfDGAT1         ---G------D----AL-----SNASSAESSHDVSFKSLVYFMVAPTLCYQPSYPRTASI309
RcDGAT1         ---E------D----A------SNSSSTEYCHDVSFKTLAYFMVAPTLCYQPSYPRTAFI308
                                                 ::   : :*  .*** *:  :*::  *

YlDGAT1         RPKHVIRNLFELVSLCMLIQFLIFQYAYPIMQSCLALFFQPKLDYANISERLMKLASVSM369
HsDGAT1         RKRFLLRRILEMLFFTQLQVGLIQQWMVPTIQNSMKPFK--DMDYSRIIERLLKLAVPNH331
MmDGAT1         RKRFLLRRVLEMLFFTQLQVGLIQQWMVPTIQNSMKPFK--DMDYSRIIERLLKLAVPNH342
NoDGAT1         RLRTLASLTFRIIVMTAFAGFIIDQQIHPIIQNTMSHVD--SLDLLKALGELLRLAIPST276
PfeDGAT1        RKGWVARQFAKQIIFTGFMGFIIEQYTNPIVRNSKHP-F--KGDLLYAIERVLKLSIPNL346
TaDGAT1         RKGWVARQFAKLVIFTGFMGFIIEQYINPIVRNSKHP-L--KGDLLYAIERVLKLSVPNL365
AtDGAT1         RKGWVARQFAKLVIFTGFMGFIIEQYINPIVRNSKHP-L--KGDLLYAIERVLKLSVPNL363
CsDGAT1         RKGWVARQFAKLVIFTGFMGFIIEQYINPIVRNSKHP-L--KGDLLYAIERVLKLSVPNL363
AhDGAT1a        RKGWVFRQLVTLIIFTGLMGFIIEQYINPIVQNSQHP-L--KGNLLYAIERVLKLSVPNL355
GhDGAT1         RKNWVVRQFIKLIIFTGLMGFIIEQYINPIVQNSQHP-L--KANFLYAIERILKLSVPNT344
VfDGAT1         RKGGVVRQFVKLIIFTGFMGFIIEQYINPIVQNSQHP-L--KGDLLYAIERVLKLSVPNL366
RcDGAT1         RKGWVFRQFVKLIIFTGFMGFIIEQYINPIVQNSQHP-L--KGDLLYAIERVLKLSVPNL365
                *   :       : :  :   :* *   * ::.        . :      .:::*:  .
```

Conserved H(X)4D/N motif in plants

Fatty acyl-CoA binding   DAG binding motif

```
YlDGAT1         MVWLIGFYAFFQNGLNLIAELTCFGNRTFYQQWWNSRSIGQYWTLWNKPVNQYFRHHVYV429
HsDGAT1         LIWLIFFYWLFHSCLNAVAELMQFGDREFYRDWWNSESVTYFWQNWNIPVHKWCIRHFYK391
MmDGAT1         LIWLIFFYWFFHSCLNAVAELLQFGDREFYRDWWNAESVTYFWQNWNIPVHKWCIRHFYK402
NoDGAT1         FVWLIFFYVYFHCTLNLFAELTRFGDRLFFKDWWNSTSFSRYWRTWNLPVHQFVVRHVYF336
PfeDGAT1        YVWLCMFYCFFHLWLNVLAELLCFADREFYKDWWNAKSVGDYWRMWNMPVHKWMVRHIYF406
TaDGAT1         YVWLCMFYCFFHLWLNILAELLCFGDREFYKDWWNAKSVGDYWRMWNMPVHKWMVRHIYF425
AtDGAT1         YVWLCMFYCFFHLWLNILAELLCFGDREFYKDWWNAKSVGDYWRMWNMPVHKWMVRHIYF423
CsDGAT1         YVWLCMFYCFFHLWLNILAELLCFGDREFYRDWWNAKSVGDYWRMWNMPVHKWMVRHIYF423
AhDGAT1a        YVWLCMFYCFFHLWLNILAELLRFGDREFYKDWWNAKTVDEYWRLWNMPVHKWMIRHLYF415
GhDGAT1         YVWLCMFYSFFHLWLNILAELLRFGDREFYKDWWNAKTVEEYWRMWNMPVHKWMVRHIYL404
```

```
VfDGAT1      YVWLCMFYCFFHLWLNILAELLRFGDREFYKDWWNARTVEEYWRMWNMPVHKWMVRHIYF426
RcDGAT1      YVWLCLFYCFFHLWLNIVAELLRFGDREFYKDWWNAKTVEEYWRMWNMPVHKWMVRHIYF425
              :**  **  *:  ** .***  *.:* *:::***: :.  :*  ** **:::  :*.*

                         Conserved catalytic histidine
YlDGAT1      PLLARGMSRFNASVVVFFFSAVIHELLVGIPTHNIIGAAFFGMMSQVPLIMATENLQHIN489
HsDGAT1      PMLRRGSSKWMARTGVFLASAFFHEYLVSVPLRMFRLWAFTGMMAQIPLAWFVGRFFQ--449
MmDGAT1      PMLRHGSSKWVARTGVFLTSAFFHEYLVSVPLRMFRLWAFTAMMAQVPLAWIVGRFFQ--460
NoDGAT1      PLLRAGASKMTANVAVFAVSAFFHELLISIPCHVVRLWAFLAMMGQIPLIYLTDQLEKTL396
PfeDGAT1     PCLRNKIPKSIAVIIAFLVSAVFHELCIAVPCRLFRLWAFMGIMFQVPLVFITNYLQE-R465
TaDGAT1      PCLRSKIPKVPAIIIAFLVSAVFHELCIAVPCRLFNLWAFMGIMFQVPLVFITNYLQE-R484
AtDGAT1      PCLRSKIPKTLAIIIAFLVSAVFHELCIAVPCRLFKLWAFLGIMFQVPLVFITNYLQE-R482
CsDGAT1      PCLRSKIPKTLAIIIAFLVSAVFHELCIAVPCRLFKLWAFIGIMFQVPLVFITNYLQE-R482
AhDGAT1a     PCIRHGVPKGVALLIAFLVSALFHELCIAVPCHIFKLWAFIGIMMQVPLVLITNYLQN-K474
GhDGAT1      PCLRNGIPKGVAILIAFLVSAIFHELCIAVPCHLFKLWAFFGIMFQAPLVLITSYLQN-K463
VfDGAT1      PCLRHKIPRGVALLITFFVSAVFHELCIAVPCHIFKLWAFIGIMFQIPLVGITNYLQN-K485
RcDGAT1      PCLRRKIPRGVAIVIAFFVSAVFHELCIAVPCHMFKLWAFFGIMFQIPLVVITNYFQR-K484
             * :     :  *   .*  **.:**  :.:* : .    ** .:* * **   .  : .

                                    ER retrieval motif
YlDGAT1      SSLGPFLGNCAFWFTF-FLGQPTCAFLYYLAYNYKQNQ------      526
HsDGAT1      ----GNYGNAAVWLS-LIIGQPIAVLMYVHDYYVLNYEAPAAEA      488
MmDGAT1      ----GNYGNAAVWVT-LIIGQPVAVLMYVHDYYVLNYDAPVGV-      498
NoDGAT1      FK-ETQAGNYTFWLIFCIFGQPMAVLLYYADFSARTTSESAL--      437
PfeDGAT1     FG--STVGNMIFWFIFCIFGQPMCVLLYYHDLMNRKGSTS----      503
TaDGAT1      FG--SMVGNMVFWFIFCIFGQPMCVLLYYHDLMNRKGSMA----      522
AtDGAT1      FG--STVGNMIFWFIFCIFGQPMCVLLYYHDLMNRKGSMS----      520
CsDGAT1      FG--STVGNMIFWFIFCIFGQPMCVLLYYHDLMNRKGSMS----      520
AhDGAT1a     FR-SSMVGNMIFWFIFCILGQPMCVLLYYHDLMSRKVKTE----      513
GhDGAT1      FQ-SSMVGNMIFWFIFCILGQPTCVLLYYHDLMNRKGSAD----      502
VfDGAT1      FR-SSMVGNMIFWFIFCILGQPMCLLLYYHDLMNRKGTTESR--      526
RcDGAT1      FR-SSMVGNMIFWFFFCILGQPMCVLLYYHDLMNRDGN------      521
                    **  .*.   ::*** . ::*
```

Supplementary figure S2: Multiple sequence alignment of DGAT2 sequences from species investigated in this study. For species names, see Supplementary data file S1.

```
YlDGA1       -MTIDSQY------------YKSRDKNDT-APKIAG-IRYAPLSTPLLNRCETFSLVWHI45
HsDGAT2      ------------------------------MDSVKGSPVTGTGSSILSALQDLFSVTWLN30
MmDGAT2      MKTLIAAYSGVLRGERRAEAARSENKNKGSALSREGSGRWGTGSSILSALQDIFSVTWLN60
NoDGTT5      -MTPQADITSKTTPNL-KTAASSPS-KTSPAPSVQYKAANGKVITVAMAEQDDGNM--GI55
RcDGAT2      -MGEEANHNNN-------------N-NNINSNDEKNEEKSNYTV---VNSRELYPT--NI40
VfDGAT2      ----------------------------MGMVEV--KNEEEVTI---FKSGEIYPT--NI25
GhDGAT2      MVEERVE-------------------------KKTYAAGSGYRE---FNGRNEFSS--NV30
AhDGAT2      -MEDRGNV--------------------TAAPPAEEKVFRSTEV---FAAESSSKS--KG34
TaDGAT2      --------------------------------------MGGSRD---FGADEHSSS--HV17
PfeDGAT2     --------------------------------------MGESRE---FQSKKAS----NQ15
AtDGAT2      --------------------------------------MGGSRE---FRAEEHS----NQ15
CsDGAT2      --------------------------------------MGGSRE---FRADKSS----DQ15
                                                                    .
```

```
YlDGA1        FS----------I----PTFLTIFMLCCAIPLL--------WPFVIAYVVYAV-KDDSPS82
HsDGAT2       RSKVEKQLQVISVLQWVLSFLVL-GVACSAILMYIFCTD-CWLIAVLYFTWLVFDWNTPK88
MmDGAT2       RSKVEKQLQVISVLQWVLSFLVL-GVACSVILMYTFCTD-CWLIAVLYFTWLAFDWNTPK118
NoDGTT5       FRECFAMVTMGIIMSWYYIVVILSLLCLVGICIFPAWRAVAATVFVLMWSAALLPLDYQG115
RcDGAT2       FHALLALS------IWIGSIHFNLFLLFISYLFLSFPTF--LLIVGFFVVLMFIPIDEHS92
VfDGAT2       FQSVLALA------IWLGSFHFILFLV-SSSIFLPFSKF--LLVIGLLLFFMVIPINDRS76
GhDGAT2       LHGILACA------MWIGALHFDFYLLVFSLLFLPFSKF--LLVVGLLLVFAVVPIDHNS82
AhDGAT2       FKTTLALA------LWLGAIHFNGALMLFALLFLPLSKA--LLVFALLFVFMVIPIDEKS86
TaDGAT2       VHSVIAIA------IWLGAIHLNVAIVLFSLIFLPPSLS--LLVFCLLFLFIFIPIPDRN69
PfeDGAT2      FHSIITMI------IWLGTIHLNVAIVLFSLIFLPPSLS--LLVFGFLSVFIFLPIDDRS67
AtDGAT2       FHSIIAMA------IWLGAIHFNVALVLCSLIFLPPSLS--LMVLGLLSLFIFIPIDHRS67
CsDGAT2       FHSTIAMA------IWLGAIHFNIVLVLFSLIFLPPYLS--LLVLSLLSLFIFIPIDHRS67
                                 .     :      :          .

YlDGA1        NGGVVKRYSPISRNFFIWKLFGRYFPITLHKTVDLEPTHTYYPLDVQEYHLIAERYWPQN142
HsDGAT2       KGGR---RSQWVRNWAVWRYFRDYF-----------------------------------110
MmDGAT2       KGGR---RSQWVRNWAVWRYFRDYF-----------------------------------140
NoDGTT5       -WDA-------FCNSFIFRLWRDYFHYEYVLE----------------------------139
RcDGAT2       KLGR-------RLCRYVCRHACSHFPVTLHVED---------------------------118
VfDGAT2       KLGQ-------CLFSYISRHVCSYFPITLHVED---------------------------102
GhDGAT2       KFGL-------RLARYICRNMSNYFPTTLHVED---------------------------108
AhDGAT2       KFGR-------KLSRYICKNACSYFPITLHVED---------------------------112
TaDGAT2       EYGR-------KLARYICKHACGYFPVTLHVED---------------------------95
PfeDGAT2      KYGR-------KLARYLCKHSCTYFPVSLHVED---------------------------93
AtDGAT2       KYGR-------KLARYICKHACNYFPVSLYVED---------------------------93
CsDGAT2       KYGR-------KLARYICKHACNYFPVSMYVED---------------------------93
                .             : :    :*

YlDGA1        KYLRAIISTIEYFLPAFMKRSLSINEQEQPAERDPLLSPVSPSSPGSQPDKWINHDSRYS202
HsDGAT2       ------------------------------------------------------------110
MmDGAT2       ------------------------------------------------------------140
NoDGTT5       ------------------------------------------------------------139
RcDGAT2       ------------------------------------------------------------118
VfDGAT2       ------------------------------------------------------------102
GhDGAT2       ------------------------------------------------------------108
AhDGAT2       ------------------------------------------------------------112
TaDGAT2       ------------------------------------------------------------95
PfeDGAT2      ------------------------------------------------------------93
AtDGAT2       ------------------------------------------------------------93
CsDGAT2       ------------------------------------------------------------93

YlDGA1        RGESSGSNGHASGSELNGNGNNGTTNRRPLSSASAGSTASDSTLLNGSLNSYANQIIGEN262
HsDGAT2       ------------------------------------------------------------110
MmDGAT2       ------------------------------------------------------------140
NoDGTT5       ------------------------------------------------------------139
RcDGAT2       ------------------------------------------------------------118
VfDGAT2       ------------------------------------------------------------102
GhDGAT2       ------------------------------------------------------------108
AhDGAT2       ------------------------------------------------------------112
TaDGAT2       ------------------------------------------------------------95
PfeDGAT2      ------------------------------------------------------------93
AtDGAT2       ------------------------------------------------------------93
CsDGAT2       ------------------------------------------------------------93

                                  PH motif
YlDGA1        DPQLSPTKLKPTGRKYIFGYHPHGIIGMGAFGGIATEGAGWSKLFPGIPVSLMTLTNNFR322
HsDGAT2       PIQLVKTHNLLTTRNYIFGYHPHGIMGLGAFCNFSTEATEVSKKFPGIRPYLATLAGNFR170
```

```
MmDGAT2       PIQLVKTHNLLTTRNYIFGYHPHGIMGLGAFCNFSTEATEVSKKFPGIRPYLATLAGNFR200
NoDGTT5       -------EMIDPNKRYLFAEMPHGIFPWGEVISIS----ITKQLFPGSRVGSIGASVIFL188
RcDGAT2       ------MNAFHSDRAYVFGYEPHSVFPLGVSVLSD-----HFAVLPLPKMKVLASNAVFR167
VfDGAT2       ------INAFRSDRAYVFGYEPHSVFPIGVMILS-------LGLIPLPNIKFLASSAVFY149
GhDGAT2       ------INYFQSDRAYVFGYEPHSVLPIGVVTLAE-----RTGFMPLPKLKCLTTSPVFY157
AhDGAT2       ------IKAFNSNRAYVFGFEPHSVLPIGVVALAD-----NTGFMPLPKIKVLASSAVFY161
TaDGAT2       ------YEAFQPNRAYVFGYEPHSVWPIGAVALAD-----LTGFMPLPNIKVLASSAVFY144
PfeDGAT2      ------YDAFQSDRAYVFGYEPHSVLPIGVVALCD-----LTGFMPLNKIKVLASSAIFY142
AtDGAT2       ------YEAFQPNRAYVFGYEPHSVLPIGVVALCD-----LTGFMPIPNIKVLASSAIFY142
CsDGAT2       ------YEAFQPNRAYVFGYEPHSVLPIGVVALCD-----LTGFMPLTKIKVLASSAIFY142
                     .     : *:*.  **.:   *               :*            *
                PR motif                             GGE motif
YlDGA1        VPLYREYLMSLGVASVSKKSCKALLKRN---QSICIVVGGAQESLLARPGVMDLVLLKRK379
HsDGAT2       MPVLREYLMSGGICPVSRDTIDYLLSKNGSGNAIIIVVGGAAESLSSMPGKNAVTLRNRK230
MmDGAT2       MPVLREYLMSGGICPVNRDTIDYLLSKNGSGNAIIIVVGGAAESLSSMPGKNAVTLKNRK260
NoDGTT5       LPGLRHFFAWIGCRPASPENIKKIFE---DGQDCAVTVGGVAEMFLVGGDKERLYLKKHK245
RcDGAT2       TPVLRHIWTWCGLTSATKKNFTALLA---SGYSCIVIPGGVQETFYMKHGSEIAFLKARR224
VfDGAT2       TPFLRHIWSWCGLTPATRKNFVSLLS---SGYSCILVPGGVQETFYMKQDSEIAFLKARR206
GhDGAT2       TPFLRHIWTWLGASPATRKNFCSLLE---AGYSCIVVPGGVQETFLMQHDSEVAFLKSRR214
AhDGAT2       TPFLRHIWTWLGLTPATKKNFLSLLD---NGYSCILIPGGVQETFLMEHGTETAYLKARK218
TaDGAT2       TPFLRHIWTWLGLAPASRKNFSSLLE---SGYSCILVPGGVQETFHMQHDVENLFLSSRR201
PfeDGAT2      TPFLRHIWSWLGLTPASRRNFTSLLD---SGYSCVLIPGGVQETYHMKHDVETVFLSSRR199
AtDGAT2       TPFLRHIWTWLGLTAASRKNFTSLLD---SGYSCVLVPGGVQETFHMQHDAENVFLSRRR199
CsDGAT2       TPFLRHIWTWLGLTPASRKNFTSLLD---SGYSCVLVPGGVQETFHMQHDVENVFLSSRR199
               *  *.     *   ..  .   ::          :  **. *      .     *  ::

                            VPFG Block
YlDGA1        GFVRLGMEVGNVALVPIMAFGENDLYDQVSNDKSSKLYRFQQFVKNFLGFTLPLMHARGV439
HsDGAT2       GFVKLALRHG-ADLVPIYSFGENEVYKQVIFEEGSWGRWVQKKFQKYIGFAPCIFHGRGL289
MmDGAT2       GFVKLALRHG-ADLVPTYSFGENEVYKQVIFEEGSWGRWVQKKFQKYIGFAPCIFHGRGL319
NoDGTT5       GFVREAMKNG-ADLVPVFCFGNSKLFNVVGESSRVSM-GLMKRLSRRIKASVLIFYGR--301
RcDGAT2       GFVRVAMEMG-KPLVPVFCFGQSNVYKWWKPDG-----ELFMKIARAIKFSPIVFWGV--276
VfDGAT2       GFIRIAMQTG-TPLVPVFCFGQMHTFKWWKPDG-----ELFMKIARAIKFTPTIFWGV--258
GhDGAT2       GFVRIAMEMG-CPLVPVFAFGQSHAYKWWKPGG-----KLILQLSRAIKFVPMLFWGA--266
AhDGAT2       GFIRIAMQKG-QPLVPVFCFGQSDIYKWWKPGG-----KLILNFARAIKFTPIYFWGI--270
TaDGAT2       GFVRIAMEHG-TPLVPVFCFGQAHVYKWWKPDW-----NLYLKLSRAIRFTPICFWGV--253
PfeDGAT2      GFVRIAMEHG-CPIVPVFCFGQSRVYSWWKPDW-----DLYLKLSRAIRFTPICFWGV--251
AtDGAT2       GFVRIAMEQG-SPLVPVFCFGQARVYKWWKPDC-----DLYLKLSRAIRFTPICFWGV--251
CsDGAT2       GFVRIAMEQG-TPLVPVFCFGQSHVYRWWKPDW-----DLYLKLSRAIRFTPICFGGV--251
              **:: .:. *   :**  .**:   :             .   . . :      : .

                             G motif
YlDGA1        FNYD-VGLVPYRRPVNIVVGSPIDLPYLPHPTDEEVSEYHDRYIAELQRIYNEHKDEYFI498
HsDGAT2       FSSDTWGLVPYSKPITTVVGEPITIPKLEHPTQQDIDLYHTMYMEALVKLFDKHKTKFGL349
MmDGAT2       FSSDTWGLVPYSKPITTVVGEPITVPKLEHPTQKDIDLYHAMYMEALVKLFDNHKTKFGL379
NoDGTT5       L----FLPIPIRHPLLFVVGKPLPVVHKAEPTKEEIAATHALFCEKVEELYYKYRPEWET357
RcDGAT2       L----GSHLPLQRPMHVVVGKPIEVKQNPQPTVEEVSEVQGQFVAALKDLFERHKARVGY332
VfDGAT2       L----GTPLPFKNPMHVVVGRPIEVKQNPQPTAEEVAEVQREFIASLKNLFERHKARVGY314
GhDGAT2       F----GTPIPYQHPMHVVVGKPIYLKKNPQPTAEEVLEVHDQFVKALEDLFERHKARVGY322
AhDGAT2       F----GSPIPFKHPMYVVVGRPIELDKNPEPTTEEVATVHSQFVASLQDLFERYKARAGY326
TaDGAT2       F----GSPLPYRHPMHVVVGKPIEVAKNLQPTNEEIAKVHSQFVEALKDMFERHKARAGY309
PfeDGAT2      F----GTPLPYREPMHVVVGKPIEVTKTLQPSDEEVAKYHGQFVEALRDLVERHKARVGY307
AtDGAT2       F----GSPLPCRQPMHVVVGKPIEVTKTLKPTDEEIAKFHGQYVEALRDLFERHKSRVGY307
CsDGAT2       F----GSPLPYRQPMHVIVGKPIEVTKTLQPTDEEIAKFHGQYVEALRDLFESHKARVGY307
              :       :*   .*:  :** *: :    .*: :::   :  :   :  :   :: .

YlDGA1        DWTEEGKGAPEFRMIE--      514
```

```
HsDGAT2         PE---------TEVLEVN     358
MmDGAT2         PE---------TEVLEVN     388
NoDGTT5         RPLSI-------E-----     363
RcDGAT2         ADLTL-------EIL---     340
VfDGAT2         SDLKL-------EIF---     322
GhDGAT2         DDLHL-------RIL---     330
AhDGAT2         PNLEL-------RIV---     334
TaDGAT2         PDLKM-------NIL---     317
PfeDGAT2        SDLQL-------KIL---     315
AtDGAT2         D-LEL-------KIL---     314
CsDGAT2         PDLQL-------NIL---     315
                            .
```

Supplementary figure S3: Chromatogram showing the HPLC separation of [14C]1HFA-DAG derived from [14C] 2HFA-TAG. Peak 1, 2, 3 represents 1HFA-DAG with HFA at either sn-1 or sn-3 position and 18:1 or 18:2 or 18:3 FA at the sn-2 position, respectively. Since, sn-1,2 and sn-1,3 fractions of each DAG molecular species are overlapping, we fraction collected all the peaks. Major peak 3 was further subjected enantiomer analysis by HPLC to confirm the proportions of *sn*-1,2 and *sn*-2,3-DAG isomers, see the figure 7 for the data.

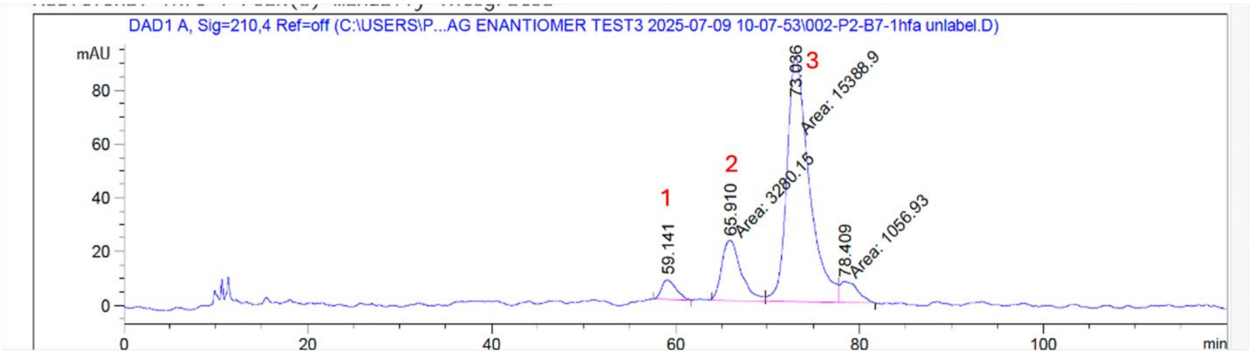

Supplement: Supplementary Figures S1-S3 [file BSR-2026-0190-T_supp.pdf]
